# Central gene transcriptional regulatory networks shaping monocyte development in bone marrow

**DOI:** 10.3389/fimmu.2022.1011279

**Published:** 2022-10-11

**Authors:** Zhaoqi Zhang, Elhusseny A. Bossila, Ling Li, Songnian Hu, Yong Zhao

**Affiliations:** ^1^ State Key Laboratory of Membrane Biology, Institute of Zoology, Chinese Academy of Sciences, Beijing, China; ^2^ University of Chinese Academy of Sciences, Beijing, China; ^3^ Biotechnology Department, Faculty of Agriculture Al-Azhar University, Cairo, Egypt; ^4^ Beijing Institute for Stem Cell and Regeneration, Beijing, China; ^5^ State Key Laboratory of Microbial Resources, Institute of Microbiology, Chinese Academy of Sciences, Beijing, China

**Keywords:** monocyte development, RNA-seq, transcriptional regulation, metabolism, molecular network

## Abstract

The development of monocytes in bone marrow is a complex process with multiple steps. We used RNA-seq data to analyze the transcriptome profiles in developing stages of monocytes, including hematopoietic stem cells (HSCs), common myeloid progenitors (CMPs), granulocyte–monocyte progenitors (GMPs), and monocytes. We found that genes related to potassium and other cation transmembrane activities and ion binding were upregulated during the differentiation of HSCs into CMPs. Protein transport and membrane surface functional molecules were significantly upregulated in the GMP stage. The CD42RAC and proteasome pathways are significantly upregulated during the development of HSCs into monocytes. Transcription factors Ank1, Runx2, Hmga2, Klf1, Nfia, and Bmyc were upregulated during the differentiation of HSCs into CMPs; Gfi1 and Hmgn2 were highly expressed during the differentiation of CMPs into GMPs; Seventeen transcription factors including Foxo1, Cdkn2d, Foxo3, Ep300, Pias1, Nfkb1, Creb1, Bcl6, Ppp3cb, Stat5b, Nfatc4, Mef2a, Stat6, Ifnar2, Irf7, Irf5, and Cebpb were identified as potentially involved in the development of GMPs into monocytes in mice and humans. In metabolism pathway regulation, HSCs have high glucose, lipid, and nucleic acid metabolism activities; CMPs mainly up regulate the TCA cycle related genes; and GMPs have extremely active metabolisms, with significantly elevated pentose phosphate pathway, TCA cycle, histidine metabolism, and purine metabolism. In the monocyte phase, the tricarboxylic acid (TCA) cycle is reduced, and the anaerobic glycolysis process becomes dominated. Overall, our studies offer the kinetics and maps of gene transcriptional expressions and cell metabolisms during monocyte development in bone marrow.

## Introduction

Monocytes/macrophages play an important role in the host’s innate immune defense and regulate adaptive immunity in many respects. The development of CD11b^+^CD115^+^Ly6G^-^ monocytes from Lin^−^Sca-1^+^c-Kit^+^CD150^+^ hematopoietic stem cells (HSCs) is a precisely regulated multiple stepwise developing process, mainly including the differentiation of HSCs into Lin^−^Sca-1^−^IL7Rα^-^c-Kit^+^CD150^−^CD34^+^FcγR^low^ common myeloid progenitors (CMPs), Lin^−^IL7Rα^-^Sca-1^−^c-Kit^+^CD150^−^CD34^+^FcγR^high^ granulocyte-monocyte progenitors (GMPs), and finally into monocytes in bone marrow in steady state (1–3). It is well known that monocyte development and survival in mice are tightly dependent on colony-stimulating factor 1 (CSF1) (4, 5). Transcription factors such as spleen focus forming virus proviral integration oncogene Sfpi1 (also known as PU.1) and CCAAT/enhancer-binding proteins (C/EBPs) play a prominent role in monocyte differentiation at various stages of commitment (3, 6). PU.1 directs HSCs to lymphoblastoid progenitor cells (LMPs) and interacts with GATA-binding protein 1 (GATA1) (7) to inhibit the differentiation of megakaryocyte-erythroid progenitor cells. C/EBPα then directs LMPs to the GMP stage while inhibiting lymphoid development by cross-repressing Pax5 and potentially other regulators (8, 9). Increased PU.1 activity favors the mononuclear cell commitment of GMPs. C/EBPα zipper with c-Jun or c-Fos also contributes to monocyte lineage specification (5). In addition, interferon regulatory factor (IRF8), Kruppel-like factor 4 (KLF4), GATA2, and Runx1 play crucial roles in the development of monocytes in bone marrow (10–12). Heterozygous familial or sporadic GATA2 mutations increase susceptibility to infection, pulmonary dysfunction, autoimmunity, lymphedema, and malignancy and gradually lose monocytes through aging (10). Accumulating studies have significantly uncovered some key molecular regulators for the differentiation of HSCs into monocytes, but we still do not have detailed insights into the roadmap of how the dynamic modulation of the gene transcription signature shapes the development of HSCs into monocytes in a whole transcriptional scale. With the emergence of big data analysis (13–15), we can now integrate all the data from multiple laboratories to analyze the metabolism and gene network changes in this development process in a more detailed, integrative way.

Given the currently available extensive transcriptomic databases from multiple laboratories all over the world and our detected RNA-seq data of mouse HSCs, CMPs, GMPs, and monocytes (13–15), we focus on the integrative analysis of transcriptome data during mouse monocyte development in bone marrow in the steady state to identify the key coordinated regulation of gene transcriptional expressions and metabolism regulatory networks in each developing stage. Considering species-specific differences, we also used the genomewide transcriptional profiling data and ATAC-seq data of human monocytes and relevant human tumor samples to confirm the key alterations of transcriptional networks and factors identified in mouse samples. We systematically investigated the dominant genetic pathways during the differentiation of HSCs into monocytes, identified “fingerprints” for each cell population and cell-type identity of developing monocyte lineage, and uncovered additional genes whose functions have been unrecognized in monocyte differentiation until now as novel candidate regulators for the certain stage of HSC differentiation into monocytes in bone marrow. The present study provided a theoretical basis and the fundamental principle for understanding the overall intrinsic transcriptional regulatory network and immunometabolism during monocyte development in bone marrow in mice and humans.

## Results

### Data processing and identification of gene transcriptional regulation modes during monocyte development

**Figure 1:**
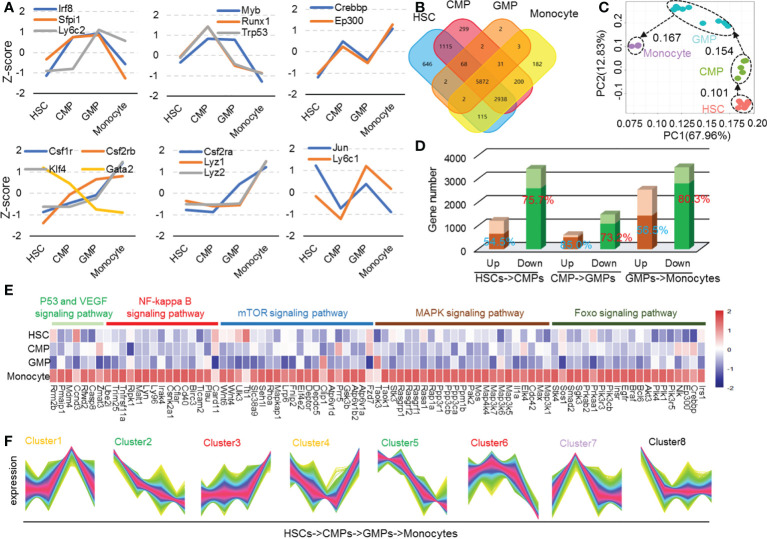
Differential gene function analysis and transcriptional regulation modes in mouse monocyte development stages. **(A)** The Z-score was used to depict the expression of conclusive molecular and transcriptional factors during mouse monocyte development. **(B)** Venn plots of mouse HSCs, CMPs, GMPs, and monocytes expression genes. The expression genes are defined as TPM > 0.1. **(C)** Population-distance analysis of genes shown in two principal components (PC1 67.96%, PC2 12.83%). Cell types are color coded, labeled by name, and surrounded by different ellipses: HSCs, red; CMPs, green; GMPs, blue; monocytes, purple. **(D)** The bar plot represents differential genetic statistics of mouse monocyte developmental phases. The bars in darker color represent the RNA-seq data measured in our own laboratory, and the entire master composed of dark and light bars represents the number of differential genes identified by the downloaded data. **(E)** Heatmap of upregulated genes in HSCs, CMPs, GMPs, and monocytes in P53, NF-kappa B, MAPK, mTOR and Foxo signaling pathways. **(F)** The line chart is the different regulation mode of expression genes in mouse monocyte development stages.

To analyze the gene transcriptional expression profile alterations during monocyte development, we first downloaded all available RNA-seq data of HSCs, CMPs, GMPs, and monocytes with C57BL/6 (B6) mouse genetic backgrounds using the well-recognized biomarkers (**Supplementary Table 1** and **Supplementary Table 2**) in NCBI and other web resources. The collected metadata were systemically analyzed as shown in **Supplementary Figure 1**. The data saturation (**Supplementary Figure 2**) and the expression abundance analysis (**Supplementary Figure 3A**) showed good quality in each cell population. Importantly, the gene expression level in each cell subset is virtually identical, as evidenced by the low standard error of each gene, although all the data were collected in different laboratories around the world (**Supplementary Figure 3B**). To further validate these metadata, we observed the expression patterns of the well-characterized key genes that mark critical molecular events in monocyte differentiation. Impressively, the transcriptional expression kinetic alterations of Lyz, Ep300, Crebbp, Csf2rb, and other well-known marker genes identified through the collected data were nicely consistent with the current reports and conclusions (**Figure 1A**) (2, 5, 16, 17). We then defined expression genes in each cell as TPM>0.1. Throughout the entire monocyte developmental process, HSCs express 10758 genes, CMPs express 10525 genes, GMPs express 5982 genes, and monocytes express 9343 genes as detected by RNA-seq (**Figure 1B**). The number of the shared genes in all these cell populations was 5872. The number of genes modulated at each cell development stage is shown in **Figure 1B**. The correlation matrix of each cell sample based on the TPM values of the expressed genes was clustered into each subpopulation as defined (**Supplementary Figure 4**). Principal components analysis shows that the gene expression profiles of all detected samples are highly correlated with the well-known cell subpopulations, and the developing routine matches well the developmental process of HSCs into monocytes (**Figure 1C**). These analyses collectively indicated the high quality of the collected metadata from all available resources.

After normalizing read counts data using DEseq2 software to reduce technical variation and to ensure that the metadata were comparable in the following assays (18), we screened upregulated (padj<0.05, log(foldchange)>0) and downregulated (q-value<0.05, log(foldchange)<0) differential genes in each monocyte developing stage with DEseq2 software. As **Figure 1D** shows, the numbers of the downregulated genes are remarkably higher than those of the upregulated genes in each stage during monocyte development in bone marrow. The more minor gene expression changes occur during the development of CMPs into GMPs (601 upregulated genes and 1483 downregulated genes), compared with gene expression profile changes during HSC differentiation into CPMs (1203 upregulated genes and 3430 downregulated genes) and GMPs to monocytes (2538 upregulated genes and 3493 downregulated genes) (**Figure 1D**). These data indicate that more dramatic gene transcriptional modulation occurs during the differentiation of HSCs into CMPs and the differentiation of GMPs into monocytes, compared with those in the differentiation of CMPs into GMPs. In addition, remarkably more gene transcriptions (total 8406 genes) were downregulated than genes upregulated (total 4347 genes) during the whole process of HSC differentiation into monocytes (**Figure 1D**), suggesting that the gene transcriptional turnoff is the major molecule alteration event during the differentiation of HSCs into monocytes in bone marrow.

To confirm the results that the altered gene expressions as concluded with the large scale of the downloaded data in the present study were reliable and reproducible, we detected the gene mRNA expression profiles using the sorted HSCs, CMPs, GMPs, and monocytes of C57BL/6 mice with the standard cell surface markers (**Supplementary Figure 5**) and RAN-seq assays (**Supplementary Table 3**). Most of the modulated genes in each cell subpopulation identified by large data assays were also altered similarly in our detected samples (**Figure 1D**, darker color bars represent genes detected by our own RNA-seq assays). Thus, in the following study, our RNA-seq assays also confirmed the unique gene profiles in each developing stage during the differentiation of HSCs into monocytes identified by the metadata analysis.

With the altered gene transcriptional expression profiles during the development of HSCs into monocytes, we analyzed whether the transcriptionally modulated genes were enriched in certain cellular components in the different developing stages using the DAVID bioinformatics resource (http://david.abcc.ncifcrf.gov/). Strikingly, the molecular locations in cellular components of transcriptionally regulated genes were located differently in different cellular components in certain developing stages of HSCs into monocytes (q-value<0.01, **Supplementary Figure 6A**). During the differentiation of HSCs into CMPs, the molecules expressed by the downregulated genes are mainly located in the cell–cell junction, actin cytoskeleton, proteinaceous extracellular matrix, membrane regions, and receptor complex; but the expressions of the upregulated genes are mainly located in centrosome, protein-DNA complex, DNA packing complex, nucleosome, and mitochondrial matrix. During the differentiation of CMPs into GMPs, the genes encoding proteins predominantly located in the cell–cell junction, actin cytoskeleton, proteinaceous extracellular matrix, membrane region, postsynapse, synaptic/postsynaptic membrane, and plasma membrane protein complex are downregulated, but the gene-encoding proteins located in the lysosome, lytic vacuole, secretory granule, external side of the plasma membrane, membrane microdomain/raft, and nuclear-ER membrane network are upregulated (**Supplementary Figure 6A**). During the differentiation of GMPs into monocytes, most downregulated genes expressed molecules that are located in ribosomal subunit, cytosolic part, Golgi apparatus part, nucleolar part, organelle membrane, outer membrane, peroxisome, oxidoreductase complex, and proteasome complex, but the upregulated genes mainly express molecules located in the adherent junction, cell–substrate junction, nuclear chromatin, and chromosomal region (**Supplementary Figure 6A**). It should be noted that many genes associated with the actin cytoskeleton and membrane region are downregulated at CMPs and GMPs but are then upregulated at monocyte stages (**Supplementary Figure 7A**). Genes expressing proteins located in lysosomes are constantly upregulated throughout the entire differentiation process of HSCs to monocytes (**Supplementary Figure 7B**), indicating that the gain of the lysosomal system, which is an important organelle for many immune functions, is a consecutive endowing process. The genes related to enzymes are upregulated in GMPs, whereas those expressing lysosomal membrane proteins are upregulated in monocytes. The transcriptionally upregulated gene expression profile may be associated with the gradual endorsement of the immune and inflammatory function by monocytes/macrophages (19).

The data were also analyzed using KEGG (http://kobas.cbi.pku.edu.cn/) pathway analysis (q-value<0.01). The results indicate that the signaling pathway changes may play complicated roles in monocyte development (**Supplementary Figure 6B**) (20, 21). The following section discusses the detailed signaling pathway changes during monocyte development. There is a significant change in the peroxisome in GMPs and ubiquitin-mediated proteolysis in monocytes (**Supplementary Figure 6B**). Signal pathways such as Rap1 and focal adhesion (22–24) are significantly downregulated during the differentiation of HSCs to CMPs. However, pathways such as PI3K-Akt, Rap1, RAS, mTOR, MAPK, NF-kB, cGMP-PKC, cAMP, calcium, and FcgR-mediated phagocytosis are upregulated in the differentiation stage of GMPs to monocytes (**Supplementary Figure 6B**). We found that the P53, VEGF, NF-kappa B, mTOR, MAPK, and FOXO signaling pathways are upregulated mainly during the differentiation of HSCs to monocytes (**Figure 1E**). Max has been reported to enhance cell proliferation, differentiation, and inflammation (25). Genes c-IAP1/2, c-FLIP, uPA, Bcl-6, Casp8, and CyclinD regulate cell survival and apoptosis (26, 27). To see the regulatory pathways of cell cycle and apoptosis during monocyte development, we analyzed the enrichment of cluster3 and cluster6 genes of cell cycle and apoptosis-related pathways shown in **Figure 1F**. The results showed that BCL6 (28), c-FLIPL, and caspase8 were elevated during monocyte development (**Figure 1F** and **Supplementary Figures 8**, **9**). Caspase 8 highly expressed in monocytes may upregulate the mitochondria-regulated apoptosis. Meanwhile, it is reported that caspase-8 is cleaved in middle or late G1 phase, while caspase-3 is activated in late G1 or early S phase (29, 30). We speculated that the upregulation of these cell cycle regulatory genes and genes such as caspase-8 also indicated the elevated G1-S cell cycle process.

**Figure 2:**
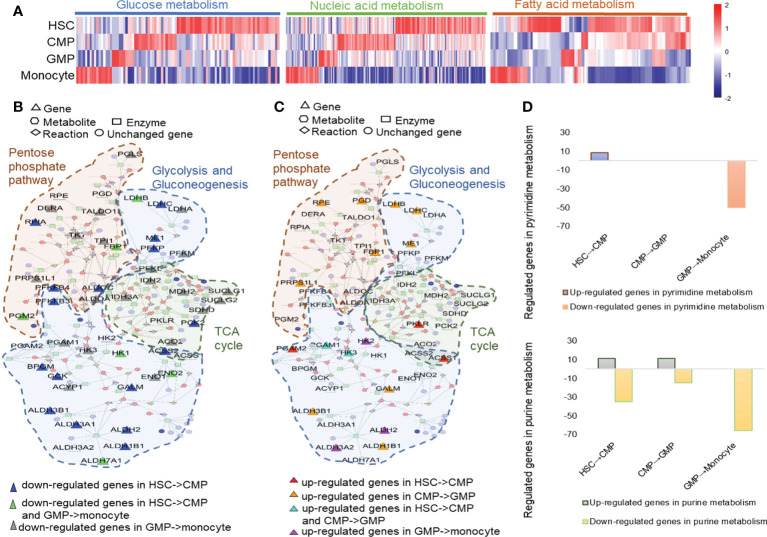
Metabolism changes during mouse HSC differentiation to monocytes. **(A)** Heatmap of glucose metabolism, nucleic acid metabolism and fatty acid metabolism-related genes based on their TPM values. The metscapes of the upregulated **(B)** and downregulated **(C)** genes in the metabolism networks of pentose phosphate pathway, glycolysis, and gluconeogenesis in mouse HSCs, CMPs, GMPs, and monocytes. **(D)** The regulated genes in pyrimidine metabolism and purine metabolism during monocyte development.

The gene transcriptional expression profile in each cell is elaborately adjusted during cell differentiation. This unique profile shapes the cell’s fate decision, differentiation direction, and functional polarization (2, 5, 31, 32). We thus performed TCseq to analyze the quantitatively and differentially regulated gene expression modes during monocyte development (33–36). The trend of gene changes during monocyte development can be divided into 8 types (highly expressed genes in HSCs, highly expressed genes in CMPs, significantly upregulated or downregulated genes in GMPs, significantly upregulated or downregulated genes in monocytes, and constantly downregulated genes) when analyzed at k=8 (**Figure 2D**). To determine whether there were genes exclusively expressed, particularly cell populations during differentiation of HSCs into monocytes in bone marrow, we then identified the highest gene transcriptional expressions in each cell population during differentiation of HSCs to monocytes. It is interesting that 1135 and 975 genes were expressed in HSCs and monocytes at the highest transcriptional levels, respectively, whereas only 122 and 112 genes were expressed in CMPs and GMPs at the highest transcriptional levels, respectively (**Supplementary Figure 10**). In contrast, 495 and 739 genes were selectively expressed in HSCs and monocytes at the lowest transcriptional levels, respectively, whereas only 125 and 65 genes were expressed in CMPs and GMPs at the lowest transcriptional levels, respectively (**Supplementary Figure 10**). More genes were expressed in HSCs and monocytes at the highest and lowest levels than in CMPs and GMPs, indicating that HSCs and monocytes are more transcriptionally active and express more widely diverse genes than CMPs and GMPs, which is consistent with the previous report (37).

### Glucose and nucleic acid metabolism modes in different stages of monocyte development

The KEGG analysis showed that metabolic changes were one of the obvious characteristics of monocyte development (**Supplementary Figure 11**). The heatmap and KEGG assays showed that the cell metabolism pathways, including glucose, nucleic acid and fatty acid metabolism, greatly shifted during HSC development into monocytes as indicated by the distinctively regulated cell metabolism-related genes in different developing cells (**Figure 2A** and **Supplementary Figure11**). With the Metscape metabolism network analysis, we found that HSCs have high levels of glucose metabolism and are composed mainly of pentose phosphate pathway, glycolysis, and gluconeogenesis (**Figure 2B**, **Supplementary Figure 11** and **Supplementary Table 6**). Genes related to glucose metabolic activity especially glycolysis and gluconeogenesis are downregulated during differentiation of HSCs to CMPs, but TCA cycle related genes, such as pyruvate kinase (Pklr), Acetate coA ligase (Acss1) were simultaneously upregulated (**Figure 2C**). Consistent with our present analysis (**Figures 2B, C**), Hannah A. Pizzato (38, 39) et al. found that HSCs usually rely on glycolysis to remain quiescent, but glycolysis process decreases and oxidative phosphorylation increases during cell differentiation. In addition to the significantly upregulated expressions of glycolytic pathway-relevant genes, such as Ldhb, Ldhc, Me1, Galm, Aldh1b1, Aldh3b1, Rrps1l1, Fbp1, Pgd and Rpe and the tricarboxylic acid cycle (TCA)-related key genes, such as succinate-CoA ligase (Suclg1 and Suclg2), succinate dehydrogenase (Sdhd), malate dehydrogenase (Mdh2) and isocitrate dehydrogenase (Idh3a), are highly expressed in GMPs, supporting that the metabolisms of pentose phosphate and aerobic glycolysis are the main metabolic pathways in GMP stage (**Figure 2C**), which is also supported by the report that the metabolism of pentose phosphate was upregulated during the cell cycle and cell proliferation (**Supplementary Figure 13**) (40). However, the genes related to the TCA cycle, such as Ilh2, Mdh2, Idh3a, Suclg1, Suclg2, Sdhd, and Aco2, are decreased, whereas genes involved in gluconeogenesis and the glycolysis process, such as Aldh2, Aldh3a2, and Hk2, are upregulated during the differentiation of GMPs into monocytes, indicating that the glycolysis process gradually shifts to the carbohydrate metabolism with the differentiation of HSCs into monocytes (**Figure 2C** and **Supplementary Figure 11**). Thus, distinctive energy supply strategies may be employed in different stages of HSCs’ development into monocytes.

Nuclear acid metabolism is also important for cell metabolism in many respects. We found that genes involved in pyrimidine metabolism and purine metabolism are significantly upregulated during the differentiation of HSCs into CMPs (**Supplementary Figure 11**). But lipid metabolism and nucleotide metabolism are significantly upregulated during the development of CMPs to GMPs (**Supplementary Figure 12**), especially histidine metabolism (Aldh3b1, Aldh1b1, and Hdc) and purine metabolism (Xdh, Atp6v1b2, Adssl1, Gda, Atp6v1a, and Atp6v1e1) pathways, which are upregulated in this process (**Supplementary Figures 11**, **14**). However, both pyrimidine metabolism and purine metabolism are significantly downregulated during the GMPs’ differentiation into monocytes (**Figure 2D**). It is well known that pyrimidines and nucleic acids form five bases of DNA and RNA and that pyrimidine metabolism is important for the synthesis of DNA and RNA. Purine is a component of nucleic acid molecules involved in the formation of purine nucleotides and a main energy form of cells (ATP and ADP). It plays an important role in many signal pathways triggered by various cell membrane receptors (cAMP and cGMP) to support cell proliferation and differentiation (41). Thus, the dynamic and distinctive alterations of nucleotide metabolisms, including purine metabolism and pyrimidine metabolism, indicate that these metabolic pathways may affect play different roles in different developing phases of monocytes.

### Aqp1 may be a potential cell surface marker for a subset of CMPs

**Figure 3:**
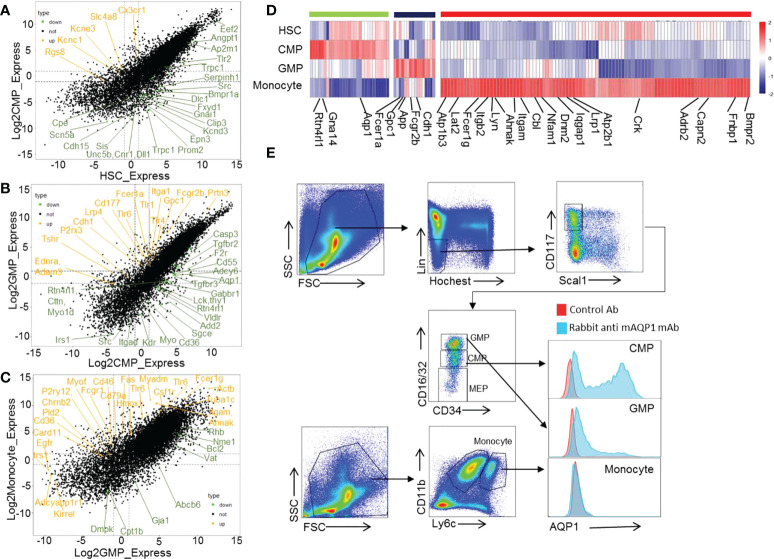
Cell membrane proteins selectively expressed in certain stage during mouse HSC differentiation to monocytes development. The dot plot showing the modulated genes between HSC–>CMP **(A)**, CMP–>GMP **(B)**, and GMP–>monocyte **(C)** during mouse monocyte development. The orange color represents the upregulated membrane-related genes, and the green color represents the downregulated membrane-related genes. **(D)** Heatmap displaying the selectively upregulated membrane-related genes in mouse HSCs, CMPs, GMPs, and monocytes. **(E)** The expression of AQP1 protein on mouse CMPs, GMPs and monocytes as detected by flow cytometry.

**Figure 4:**
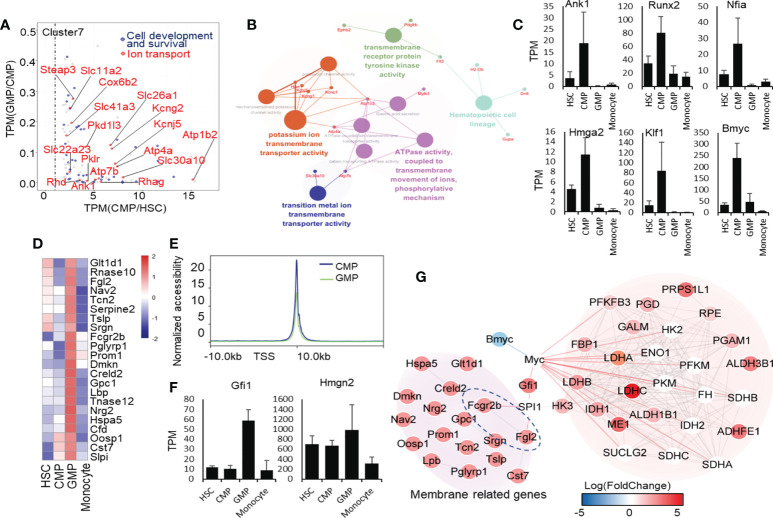
Transcriptional regulatory signatures in CMP and GMP developmental stages. **(A)** Dot plot of CMP highly expressed genes. The red color indicates the genes enriched in ion transport, and the blue color indicates the genes enriched in cell development and survival. **(B)** Network of ion-transport–related genes, which are highly expressed in mouse CMPs. **(C)** Selectively high expression of transcription factors in the CMP stage. **(D)** Heatmap of genes related to extracellular region part (GO:0005576). **(E)** The line chart shows the average intensity of TSS in mouse GMPs and CMPs. **(F)** High expressions of transcription factors selectively in the GMP stage. **(G)** Molecular network of membrane receptor-TF-metabolism regulated network.

To identify the potential new cell surface biomarkers for certain cell subsets during HSC differentiation into monocytes, we collected the membrane genes that are highly expressed in certain cell subsets by gene ontology (GO) analysis and then selected the genes expressed on the cell surface (**Figures 3A–C**). We found that transport cytoskeleton and ATPase genes (Ap2a1, Ptk2b, Ap2m1, and Slc2a3), ion channel-related genes (Atp1a1, and Gabrr1), and G protein-related genes (Gnai3, Tnk2, and Gnb4) are highly expressed in HSCs (**Supplementary Figure 15A**). The energy production-related and protein transport-related genes (Aqp1, Gna14, and Rtn4rl1) are selectively and highly expressed in CMPs (**Figure 3D**). Aqp1 is a molecular water channel and non-selective cation channel protein. Gna14 (Guanine nucleotide-binding protein subunit alpha-14) serves as modulators or transducers in various transmembrane signaling systems (42). Thus, these genes may play a role in CMP differentiation, which must be demonstrated with genetic approaches. We identified genes Cdh1, App, and Gpc1 expressing proteins located on the cell surface membrane that are highly expressed in GMPs (**Figure 3D**). The membrane-related genes Atp1b3, Lat2, Fcer1g, Lyn, Itgb2, Ahnak, Itgam, Cbi, Nfam1, Dnm2, Iqgap1, Lrp1, Atp2b1, Crk, Adrb2, Capn2, Fnbp1, and Bmpr2 are highly expressed in monocytes. These results and regulated genes in certain developing stages of monocytes were verified by our RNA-seq data (**Supplementary Figure 15B**). Considering the shortage of specific biomarkers for CMPs, we thus checked the protein expressions of these molecules on CMPs using commercially available mAbs. But we only got the antibody for mouse Aqp1 to allow us to detect the protein expression of Aqp1 on the cell surface. As shown in **Figure 4E**, Aqp1 protein is selectively expressed on a fraction of CMPs and then downregulated in GMPs, whereas monocytes are negative for Aqp1 expression as determined by flow cytometry (**Figure 3E**). However, there was no difference in the differentiation function of Aqp1^+^CMPs and Aqp1^-^CMPs (**Supplementary Figures 16A–C**). At the same time, we sorted CMPs with high Aqp1 expression and cultured them with MCSF (25ng/ml) for 0,1,2 and 4 days. And found that the expression of Aqp1 gradually decreased with the cell development process (**Supplementary Figure 16D**). Thus, our sequencing data and flow cytometry data demonstrated that Aqp1 is highly expressed in CMPs but is gradually downregulated and finally turned off in monocytes.

### Gene transcriptional characterization of CMPs and GMPs

We analyzed the gene transcriptional expressions highly regulated in CMPs (cluster 7, **Figure 1F**). Through KEGG analysis, we found that the genes highly expressed in CMPs were significantly enriched in the ion binding as well as hemopoiesis (**Figure 4A**; **Supplementary Figure 17**). We found that the potassium-binding and other ion transport genes were upregulated in CMPs by GO enrichment analysis. We then used these upregulated ion transport-related genes in CMPs for molecular network analysis and found that they predominantly regulated multiple pathways such as integrin binding, proliferation, extracellular matrix organization, and ATPase activity (**Figure 4B**). Fms-related tyrosine kinase 3 (FLT3) is a cell surface receptor expressed by various hematopoietic progenitor cells (43). From the network, we found that FLT3 is also expressed in the premature progenitor cells of the myeloid lineage and is highly expressed in the CMP stage (**Figure 4B**), which regulates the hematopoietic cell lineage as a transmembrane receptor protein. In addition, the high expressions of Atp1b2 and Atp4a genes in the CMP stage (**Figure 4B**) may regulate ion transport by ATPase-coupled ions transmembrane movement. Thus, the significantly upregulated potassium and other cation transmembrane activities in CMPs likely drive the differentiation of HSCs into CMPs, which needs confirmation through genetic approaches in animal models.

However, we like to identify the key transcription factors that may predominantly regulate the pathway shifts in CMPs, using the ENCODE database of NetworkAnalyst (44, 45) with threshold criteria, including p-value<0.05 and human homologous genes. We identified 6 highly expressed transcription factors in CMPs (**Figure 4C**). Among the transcription factors specifically expressed in the CMP stage, Nfia and Klf1 have been reported as positively correlated with MegE lineage (46) and play an important role in erythroblast enucleation (47, 48). Overexpression of Nfia in human bone marrow progenitor cells attenuates monocyte and granulocyte differentiation (49). Hmga2 has been reported to promote long-term engraftment and myeloid erythroid differentiation of human hematopoietic stem and progenitor cells (50). Moreover, we found that transcription factors Runx2, Nfia, Bmyc, and Klf1, highly expressed in CMPs, are closely associated with ion transport genes (51–55). Through String network analysis (**Supplementary Figure 18**), we concluded that the transcription factors Ank1, Runx2, Nfia, Bmyc, and Klf1 may regulate CMP development through the regulation of the ion transport network. Surprisingly, we have discovered Bmyc as a new potential transcription factor to control the differentiation of HSCs into CMPs, which has not been reported so far. Bmyc is a member of the Myc transcriptional regulator family in mice and has been reported as a transcription factor inhibiting Myc *in vitro* (56). By analyzing scATAC-seq data of human HSCs, CMPs, GMPs and monocytes, we found that the Runx2 and Hmga2 in mice identified by our analysis also showed the similar expression trend in human scATAC-seq (**Supplementary Figure 19**) (57).Nevertheless, the functions of these newly identified transcription factors in CMPs are worthy of future exploration.

To uncover the modulating characteristics of the gene transcriptional expression networks during the differentiation of CMPs into GMPs, we collected those genes transcriptionally regulated during the differentiation of CMPs into GMPs for further analysis (**Supplementary Figure 20A**). After analyzing the functions of genes downregulated during the differentiation of CMPs into GMPs, we found that these downregulated genes are enriched in GTPase-related functions (**Supplementary Figures 20B, C**). KEGG and GO functional analysis of genes upregulated in GMPs showed that genes related to the vesicle, extracellular region, protein process, infection, and metabolism are positively regulated during the differentiation of CMPs to GMPs (**Supplementary Figures 21A, B**). Many of the extracellular protein-related genes and membrane receptor genes, including Slpi, Cst7, Oosp1, Cfd, Hspa5, Rnase12, Rnase10, Lbp, Gpc1, Creld2, Dmkn, Prom1, Pglyrp1, Fcgr2b, Srgn, Tslp, Nav2, Fgl2, and Glt1d1, were highly expressed in GMPs (**Figure 4D**), which means that extracellular secretion, intracellular vesicle transport, and intercellular communication are enhanced in the GMP phase. The ATAC-seq results showed that the average accessibility intensity of transcription start site (TSS) in GMPs is lower than that of CMPs, indicating that the overall chromatin accessibility is lower in GMPs than in CMPs (**Supplementary Table 4**, **Figure 4E**), which is consistent with the RNA-seq analysis data showing that more genes are downregulated during the development of CMPs into GMPs (**Figure 1F**).

In identifying which transcription factors play a major role in the differentiation of CMPs into GMPs, we found that only two transcription factors are highly expressed in GMPs (**Figure 4F**). High mobility family nucleosome binding domain 2 (HMGN2) is a small unique non-histone protein and has many biological functions, including chromatin structure, regulation of transcription, and DNA repair (58), which may affect the nucleotide metabolism (**Figure 4F**). We further analyzed the correlation between the elevation of membrane proteins with the potential downstream transcription factors and metabolism during the developing process CMPs to GMPs. Through protein-protein interaction network analysis, we found that Fcgr2b and Fgl2, which are highly expressed in GMPs (**Figure 4D**), may be one of key receptors to stimulate the transcriptional expression of Gfi1 through Spi1 (**Figure 4G**) (59). It is reported that Gfi1 has a positive regulatory effect on Myc and then regulates the key glycolysis and lactate metabolic enzymes, such as Ldha, Ldhb, and Idh1 (59). Therefore, we speculate that the activation of Fcgr2b and Fgl2 receptors regulates transcription factor Gfi1 and Myc expressions to increase glycolysis and lactate metabolism during the development of CMPs to GMPs.

### Positively correlated genes during monocyte development

**Figure 5:**
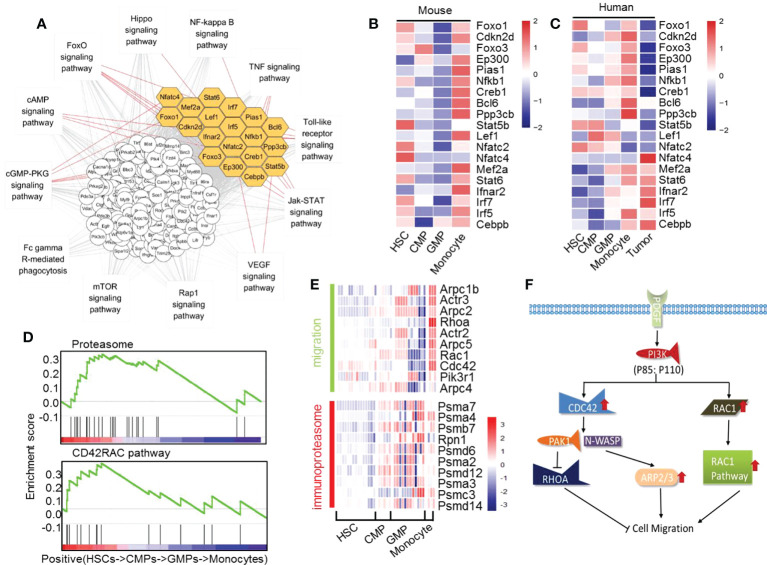
The significantly upregulated genes in mouse monocyte development. **(A)** Interaction network of the upregulated transcription factors and pathways in the differentiation of GMPs to monocytes. Yellow indicates the transcription factors. **(B)** Heatmap of the upregulated transcription factors during differentiation of GMPs into monocytes. **(C)** Heatmap of transcript factors changes in mouse and human HSCs into CMPs, CMPs into GMPs, and GMPs into monocytes, including human tumor differently expressed transcription factors. **(D)** GSEA quasi-time series analyses of genes related to CD42RAC pathway and proteasome pathway in HSCs, CMPs, GMPs, and monocytes. **(E)** Heatmap of migration-related genes and immunoproteasome-related genes during mouse monocyte development. **(F)** Proposed model of the PI3K, CDC42, and RAC1 pathways related to the cell migration during monocyte differentiation.

With these series analyses, we found that many signal pathways are obviously upregulated and downregulated during GMP differentiation into monocytes (**Supplementary Figure 22A** and **Supplementary Figure 23**). We then identified which transcription factors may play an important role in the differentiation of GMPs into monocytes. Analysis of transcription factors and regulatory gene networks revealed 19 distinct transcription factors in the pathways that are significantly upregulated during development of GMPs into monocytes (**Supplementary Figure 22B**). Inflammation, cell migration, and proteasome are interdependently affected by networks of signaling pathways through many transcription factors, including Foxo1, Cdkn2d, Foxo3, Ep300, Pias1, Nfkb1, Creb1, Bcl6, Ppp3cb, Stat5b, Lef1, Nfatc2, Nfatc4, Mef2a, Stat6, Ifnar2, Irf7, Irf5, and Cebpb during the development of GMPs into monocytes (**Figures 5A, B**). By analyzing with human scATAC-seq data of HSCs, CMPs, GMPs and monocytes, we found that the transcription factors Nfkb1, Ep300, Foxo1, Cebpb, Irf5, Mef2a, Irf7, Stat6 and Creb1 also showed the similar changing trend during the development of GMPs into monocytes (**Supplementary Figure 24**) (57). Importantly, a comparison of the key regulated transcription factors during GMP differentiation into monocytes found that most of these genes (17/19) show consistent upregulated expression tendency in mice and humans, except for Nfatc2 and Lef1, which are decreased in human monocytes (**Figure 5C**). These data support that these key transcription factors and regulatory networks in monocytes are highly conservative across species.

To investigate whether the major transcriptional regulation of those genes in mouse monocyte development is also involved in human monocyte development, we downloaded RNA-seq data from tumor cells of patients with acute monocyte leukemia to analyze the expressions of these transcription factors compared with the healthy monocytes (**Supplementary Table 5**). We found that the expressions of these transcription factors in patients with acute monocyte leukemia are significantly different in healthy people. The transcript factors Foxo1, Cdkn2d, Foxo3, Ep300, Pias1, Nfkb1, Creb1, Bcl6, Ppp3cb, Mef2a, and Irf5 are lower in acute monocyte leukemia than those of normal human monocytes but that the transcript factor Nfat4, Ifnar2, Irf7, and Cebpb are higher in acute monocyte leukemia than those of normal human monocytes (**Figure 5C**). These results further indicate that the transcription factors Foxo1, Cdkn2d, Foxo3, Ep300, Pias1, Nfkb1, Creb1, Bcl6, Ppp3cb, Stat5b, Nfatc4, Mef2a, Stat6, Ifnar2, Irf7, Irf5, and Cebpb are potential key factors mastering the normal differentiation of GMPs into monocytes in mice and humans. This speculation needs confirmation through future experimental studies.

The genes that are transcriptionally modified in either positive way during the differentiation of GMPs into monocytes are summarized in **Figure 5D** and **Supplementary Figure 23B**. It was reported that the extensive network of cytoskeletal and extracellular matrix proteins increases during the developmental progression of myeloid precursor cells, which may promote adhesion and chemotaxis in pluripotent states (60). Using time series analysis of gene set enrichment analysis (GSEA), we found that the pathways that master cell migration and proteasome are significantly upregulated during GMP differentiation into monocytes (**Figures 5D, E**), which may be beneficial for the maturing monocytes to migrate from bone marrow to the peripheral blood. The upregulated migration-relevant genes in monocytes such as Arpc1b, Arpc2, Arpc5, Rac, and PI3K would increase the migration ability of monocytes (**Figures 5D, E**). Meanwhile, the upregulated PDFGR-PI3K-CDC42/Rac1-Arp pathway (**Figures 5D, F**) would increase fiber focal adhesion, filopodium, and lamellipodium to allow monocytes to gradually acquire migration ability. This speculation was nicely supported by experimental studies showing that the PDFGR-Pi3k-CDC42/Rac1-Arp pathway is involved in the migration of macrophages (61, 62). The ubiquitin-proteasome pathway plays an important role in various basic cellular processes. In particular, it plays a key role in short-lived and regulatory protein degradation, including regulation of the cell cycle, cell surface receptors, ion channels, and antigen presentation (63). Obviously, proteasome complex-related genes such as POMP, PA28a, PA28β, and other genes are significantly and gradually upregulated during the whole differentiation process of HSCs into monocytes (**Figures 5D, E**). These upregulated genes would greatly help form immunoproteasome in monocytes, which may subsequently increase the monocytes’ shearing of the antigen (63).

### Negatively correlated genes during monocyte development

**Figure 6:**
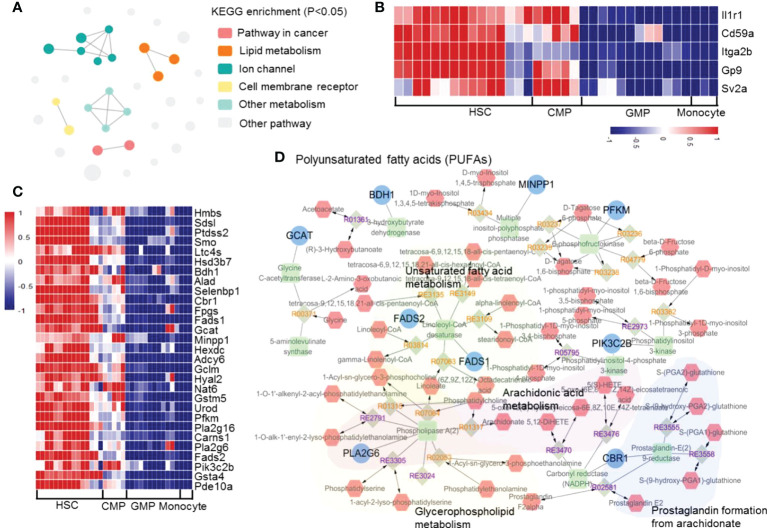
The significantly downregulated genes in mouse monocyte development. **(A)** Enrichment network of the upregulated transcription factors and pathways in the differentiation of HSCs into monocytes. Each node represents an enriched term, and the node color represents different clusters; the node size represents 6 levels of enriched p-values. **(B)** Heatmap of the downregulated genes in hematopoietic cell lineage pathway. **(C)** Heatmap of the downregulated genes in lipid metabolism-related pathways. **(D)** Network of consistently downregulated genes enriched in metabolism pathways.

We analyzed the gene transcriptional expressions that are significantly downregulated during the differentiation of HSCs into monocytes. According to the KEGG enrichment p-value, we divided the enrichment results into 6 clusters (KOBAS: http://kobas.cbi.pku.edu.cn/genelist/). The results showed that two functionally related pathways, membrane surface receptors (Il1r1, Cd59a, Itga2b, Gp9 and Sv2a) and lipid metabolism, are significantly reduced during the development of HSCs into monocytes (**Figures 6A, B** and **Supplementary Figure 25**). It has been reported that these continuously downregulated genes are associated with other hematopoietic cell lineage differentiation. For example, Il1r1 has been reported to control neutrophil development (64), CD59 plays an important role in erythrocyte development (65), and CD41 and CD42 may affect platelets development (66). Downregulation of these genes tightly controls the development of HSCs into monocytes and blocks the differentiation into other cell lineages. On the other hand, we found that some of the metabolism-related genes are downregulated during monocyte development (**Figure 6C**). We used Metscape to enrich the metabolism-related genes and found that the genes related to unsaturated fatty acid metabolism, arachidonic acid metabolism, glycerophospholipid metabolism, and prostaglandin formation from arachidonate pathways are downregulated during monocyte development (**Figure 6D**). These results suggested that the shutdown of polyunsaturated fatty acids (PUFAs) might be involved in monocyte development.

## Discussion

In the present study, with the RNA-seq data of mouse and human HSCs, CMPs, GMPs, and monocytes from the current NCBI and our own transcriptome sequencing of the sorted cells, we thoroughly analyzed the relationship between gene transcriptional networks and the development stages of monocytes, based on the high homology of mouse and human genomes (67). With almost 15,000 identified expressing genes, including more than 3,000 significantly regulated genes, these data represent a deep analysis comparing HSCs, CMPs, GMPs, and monocytes. Many genes and gene clusters are downregulated during the differentiation of HSCs to CMPs, which may be related to the fact that the genes related to cell stemness have been shut down during monocyte development (68). We found that the ion-binding–related genes are significantly upregulated from HSCs to CMPs. The CD42RAC pathway and the immunoproteasome pathway are consistently upregulated during monocyte development to promote the migration of early developing monocytes from bone marrow to the periphery and development of antigen-presenting functions. The extremely small number of genes upregulated throughout the developmental phase of HSCs to monocytes suggests that monocyte development may not be determined by a specific number of genes across the whole process; instead, each developmental phase may require different transcriptional regulation modes during the differentiation of HSCs to monocytes.

**Figure 7:**
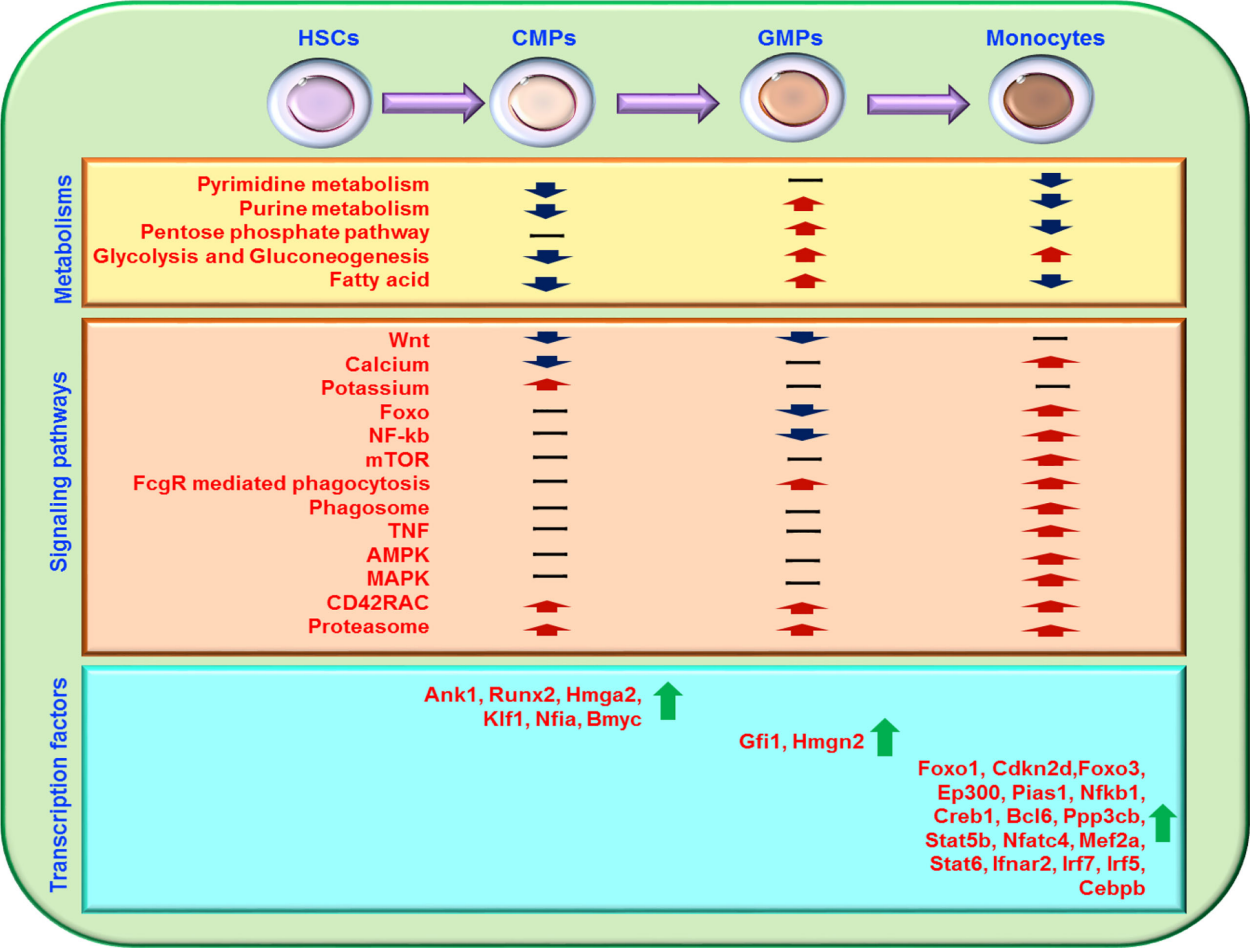
Schematic diagram of the major transcriptional regulation during the development of HSCs to monocytes. Most gene transcriptional expressions related to cell metabolisms, signal pathways, and transcription factors during the differentiation of HSCs into monocytes are summarized. “-” represents no significant regulation, “↑” represents upregulation, and “↓” represents downregulation.

Potassium-related pathways are upregulated during the differentiation of HSCs to CMPs. The Wnt, Foxo, NF-kb, mTOR, and PI3K-Akt signaling pathways decrease significantly in the CMP differentiation into GMP phase, whereas FcgR-mediated phagocytosis, phagosome, Phospholipase D, Foxo, PI3K-Akt, calcium, TNF, VEGF, mTOR, AMPK, MAPK, and lysosome pathways are upregulated in the developing phase of differentiation of GMPs into monocytes (**Figure 7**). During the development of CMPs into GMPs, cell membrane-associated genes, especially those related to cell transport function, are upregulated, indicating that the vesicle transport function of cells was significantly increased in this differentiation phase. In addition, we have analyzed transcriptome data at various stages of monocyte development. We can convert the transcription of monocyte development into eight patterns. Through sequencing data, we found that membrane-related genes Aqp1, Gna14, and Rtn4rl1 are selectively and highly expressed in CMPs, whereas Cdh1, App, and Gpc1 are highly expressed in GMPs. With flow cytometry analysis, we confirm that Aqp1 was selectively expressed on the cell surface of CMPs, which may serve as a surface marker for CMPs in mice. The membrane-related genes Atp1b3, Lat2, Fcer1g, Lyn, Itgb2, Ahnak, Itgam, Cbi, Nfam1, Dnm2, Iqgap1, Lrp1, Atp2b1, Crk, Adrb2, Capn2, Fnbp1, and Bmpr2 are highly and selectively expressed in monocytes. The biological functions of these genes in monocytes must be addressed in the future. The transport and secretory function are significantly increased, and phagosome begins to increase at the stage at which CMPs develop into GMPs. Thus, the diverse immune function of monocytes is gained in different differentiation kinetics during differentiation.

Metabolism regulates monocyte development in many ways. Glucose, lipid, and nucleotide metabolisms are at a high level in HSCs (69) (**Figure 7**). Our analysis showed that HSCs have high levels of glucose metabolism and are composed mainly of pentose glycolysis, and gluconeogenesis, whereas CMPs downregulate these pathways but upregulate genes in the TCA cycle pathway. The cell metabolism of GMPs is extremely active, as indicated by the significantly elevated pentose phosphate pathway, lipid metabolism, and nucleotide metabolism in GMPs. In the monocyte phase, the TCA cycle is reduced, and the anaerobic glycolysis process becomes dominated, indicating that the glycolysis process gradually shifts to the pentose phosphate metabolism with the differentiation of HSCs into monocytes.

Nuclear acid metabolism is also important for cell metabolism in many respects. The genes involved in pyrimidine metabolism and purine metabolism are significantly upregulated during the differentiation of HSCs into CMPs, and the lipid metabolism and nucleotide metabolism are significantly upregulated during the development of CMPs into GMPs. However, both pyrimidine metabolism and purine metabolism are significantly downregulated during the differentiation of GMPs into monocytes. The alterations of nucleotide metabolism indicate that these metabolic pathways may play different roles in different developing phases of monocytes.

Different transcription factors play different important roles in different stages of monocyte development (**Figure 7**). Our analysis showed that Ank1, Runx2, Nfia, Hmga2, Klf1, and Bmyc are more highly expressed in CMPs than in HSCs, suggesting that these transcription factors may be involved in shaping the development of HSCs into CMPs. By comparison with human scATAC-seq data, we found that most of the stage-specific transcription factors in mice we analyzed also showed the same trend in human HSCs, CMPs, GMPs and monocytes scATAC-seq (57). Fewer transcription factors are upregulated in the development of CMPs into GMPs, indicating that post-transcriptional regulatory systems may drive cell development in this stage. But we found that the regulation of Fcgr2b/Fgl2-Gfi1-Myc pathway may play an important role in CMPs to GMPs. Because RNA-seq analysis is performed at the transcriptional level and changes in post-transcriptional modification or protein modification cannot be directly detected in the present study, the roles of the nontranscriptional regulation in this process require investigation. With the analysis of mouse and human’s RNA-seq data, we found the transcriptional expressions of transcription factors Foxo1, Cdkn2d, Foxo3, Ep300, Pias1, Nfkb1, Creb1, Bcl6, Ppp3cb, Stat5b, Nfatc4, Mef2a, Stat6, Ifnar2, Irf7, Irf5, and Cebpb are consistently increased in the development of human and mouse GMPs into monocytes. By analyzing the RNA-seq data of acute monocytic leukemia, we found that the transcriptional expression of the transcription factors Foxo1, Cdkn2d, Foxo3, Ep300, Pias1, Nfkb1, Creb1, Bcl6 and Ppp3cb are reversely downregulated in acute monocytic leukemia compared with monocytes in healthy individuals. And it is reported that Foxo1 (70) and Cdkn2d (71) activity is critical for the maintenance of leukemia. Forkhead box proteins are a group of transcriptional factors implicated in different cellular functions such as differentiation, proliferation and senescence (72) to affect hematopoietic tumor, while EP300 suppresses leukemia development in myelodysplastic syndromes through inhibiting Myb (73). It has also been reported that mutations in the human Ank1 (74, 75), Runx2 (76–79), Nfia (80), Hmga2 (81), and Klf1 (82, 83) genes caused diseases of the hematopoietic system, such as hereditary spherocytosis, fetal anemia, myelodysplastic syndrome, and acute erythroleukemia. The diseases caused by mutations in these genes strongly support our speculation that the transcription factors Ank1, Runx2, Nfia, Hmga2, Klf1, and Bmyc may play important roles in monocyte development. Thus, these newly identified transcription factors may play important roles in monocyte development in bone marrow, which is worthy of future study.

Our present studies use the combined all existing RNA-seq data of HSCs, CMPs, GMPs, and monocytes from different laboratories throughout the world to perform integrative big data analysis to perform differential gene analysis using the DEseq2 application. We found that more than 80% of the differential genes obtained by integrating the downloaded data were consistent with the data measured in our laboratory, supporting the reliability and reproducibility of this analysis approach. That more differential genes were identified in the combined data than in our own detected data indicates that the analysis with the combined data was more sensitive than that with single lab data, which was possibly due to the increased sample number. We also know that pre-mRNA splicing is a critical step in gene expression that results in the removal of intron sequences from immature mRNA, resulting in mature mRNA that can be translated into protein. In order to explore whether the changes at the metabolic and transcriptional levels we found are related to RNA splicing, we performed RNA splicing analysis on the raw data by rmats software, and the results showed that the specific changes in the pathway process we found may be weakly related to RNA splicing (Supplementary material-RNA splicing analysis).

In summary, we offered a more comprehensive gene transcriptional expression profile and regulatory networks for the differentiation of HSCs into monocytes in mice. We also identified the potential key transcription factors for certain differentiation stages in the whole monocyte development process. We believe that systemic analysis of RNA-seq data with other big data would provide a fundamental molecular regulatory map for our comprehensive understanding of the effect of transcriptional regulation on mononuclear cell lineage development.

## Materials and methods

### Mice

C57BL/6 (B6) mice were purchased from the Beijing University Experimental Animal Center (Beijing, China). All mice were bred and maintained in specific pathogen-free conditions. All experimental manipulations were undertaken in accordance with the Institutional Guidelines for the Care and Use of Laboratory Animals, Institute of Zoology, Chinese Academy of Sciences.

### Antibodies and flow cytometry

Antibodies to the following were purchased from Biolegend, eBioscience and Bioss and were used at 1:100-1:400 dilutions: B220-FITC, CD11b-FITC, CD4-FITC, CD8-FITC, TER-119-FITC, Gr-1-FITC, SCAL-1-APC-CY7, SCAL-1-percpcy5.5, C-KIT-PE-CY7, C-KIT-APC, CD16/32-PRE-CY5, CD16/32-PE, CD150-PE, CD34-Alexa Fluor^®^700, CD11b-PE-CY5, CD45-PE-CY7, CD115-PE, LY6G-FITC, rabbit anti-mouse AQP1, and goat anti-rabbit 488. For flow cytometric analysis of surface markers, cells were stained with antibodies in PBS containing 0.1% (w/v) BSA and 0.1% NaN_3_. For the myeloid progenitors isolated method, lineage cells were isolated by the negative selection procedure of magnetic-activated cell sorting using MS Lineage Panel Biotin (Biolegend) and BD beads (#559971). MoFlo™ XDP (Beckman) was used to sort HSC (Lin^−^Sca-1^+^c-Kit^+^CD150^+^), CMP (Lin^−^Sca-1^−^c-Kit^+^CD150^−^CD34^+^FcγR^low^), and GMP (Lin^−^Sca-1^−^c-Kit^+^CD150^−^CD34^+^FcγR^high^) cells. Biotin-CD3e antibody, biotin-TER119 antibody, biotin-CD45R antibody, and biotin-Ly6G antibody were used with magnetic-activated cell sorting to remove T cells, B cells, and granular cells, followed by MoFlo™ XDP (Beckman) to sort monocytes (CD11b^+^CD45^high^CD115^+^Ly6G^-^).

### RNA-seq and ATAC-seq data collection

We used the existing secondary generation sequencing RNA-seq and ATAC-seq results from the SRA database, which was specifically developed to collect RNA-seq and ATAC-seq data, to perform deep analyze the transcriptional profile alteration during monocyte development in bone marrow. To ensure that these data were comparable with ours, we used HSCs (Lin^−^Sca-1^+^c-Kit^+^CD150^+^), CMPs (Lin^−^Sca-1^−^c-Kit^+^CD150^−^CD34^+^FcγR^low^), GMPs (Lin^−^Sca-1^−^c-Kit^+^CD150^−^CD34^+^FcγR^high^), and monocytes (CD11b^+^CD45^high^CD115^+^Ly6G^-^) of B6 mice to perform RNA-seq data assays.

### Genomic and annotation information data

We downloaded the mouse reference genomic sequence GRCm38 (mm10) from the UCSC database and obtained mouse gene annotation information from NCBI’s RefSeq data, Ensembl, and UCSC databases.

### RNA-seq raw data processing

The raw data downloaded from the database is stored in fastq format and contains sequencing quality and sequence information. We used Perl design procedures to complete the two-filtering work. The processing of the original data consisted of two aspects: first, filtering the low-quality reads (Q<20) and, second, the Solexa platform designs a specific sequence link at the 5’ end of each sequence at the time of library preparation. These joints do not exist in the original sequence but can be read out in the sequencing; therefore, they must be filtered to remove the linker sequence. Filtering the raw data results in high-quality read data. To screen out the search data that is positioned on the genome, we mapped the read data to the mouse mm10 reference genome using HISAT2 mapping software (84).

### RNA-seq data saturation analysis

Based on the total number of readings, we measured 10-14 reads (%) randomly between 0% and 100% of the RNA-seq reads. They were then mapped to the mouse genome and the number of corresponding expression genes was examined. The number of comparative genes is stabilized when the proportional values are randomly selected. RNA-seq data saturation analysis can determine whether the transcription amount of each sample can be saturated. The selected qualified data were used for comparative analysis of subsequent gene expression profiles.

### Extract gene expression data and differential gene analysis

Select String-tie software was used to construct transcripts independently for each cell (the mapping result file for each cell was submitted to String-tie) (84). The gene expression kurtosis map was drawn using the R language editor. A comparison was made of the coincidence of the trend of expression of RNA-seq data in different cells.

DEseq2 software was used to identify the differentially expressed genes of HSC–CMP, CMP–GMP, and GMP–Monocytes (18). We first normalized the data and eliminated the batch effect. Then, we set p <= 0.05 and log2(foldchange) to a significant difference in the two cell thresholds. We deleted the gene that was not fully expressed, thus ensuring that the resulting difference gene was truly different rather than caused by inter-laboratory measurement errors. The R language editing program was used, according to the screening of differences in genes and genetic trends, to draw the thermal spectrum and volcanic map and the visual display of individual genes in different cell expression trends.

### Gene ontology, KEGG pathway, and gene network analysis

GO functional annotation analysis was performed on all cell differential genes using the DAVID Bioinformatics Resources 6.8 online search tool (https://david.ncifcrf.gov/) (85, 86). KEGG pathway analysis was performed on all different genes of each cell using the KOBAS online search tool (http://kobas.cbi.pku.edu.cn/) (87, 88). The KEGG analysis results were used to analyze the metabolic pathways, visualized by iPath 3 (https://pathways.embl.de/ipath3.cgi) and Cytoscape software, using NetworkAnalyst (http://www.networkanalyst.ca/faces/home.xhtml) to find the network of proteins and transcription factors of the target gene (44, 45, 89–92). This network was then imported into Cytoscape for visualization.

### ATAC-seq analysis

Peaks of ATAC-seq were generated by macs2 v2.1.1 (93) with “-g mm –nomodel –shif -50 –extsize 100” and peak regions were merged based on their overlap using bedtools, which also calculated RPKM with multicopy. Normalized (RPKM) ATAC-seq profile for every regulatory element was calculated by segmenting a ± 10,000 bp window around its TSS using Deeptools.

## Data availability statement

The datasets presented in this study can be found in online repositories. The names of the repository/repositories and accession number(s) can be found in the article/**Supplementary Material**.

## Ethics statement

The animal study was reviewed and approved by the Institutional Guidelines for the Care and Use of Laboratory Animals, Institute of Zoology, Chinese Academy of Sciences.

## Author contributions

ZZ, EB, and LL collected and analyzed the data and interpreted the results. ZZ performed the experiments. ZZ and YZ drafted the article. SH critically revised the article. SH and YZ supervised the project and designed the experiments. All authors contributed to the article and approved the submitted version.

## Acknowledgments

The authors thank Drs. Peng Wang and Zhanfeng Liang for their critical reading of the manuscript. This work was supported by grants from the National Natural Science Foundation for General and Key Programs (31930041, YZ), the National Key Research and Development Program of China (2017YFA0105002, 2017YFA0104402, YZ), and the Knowledge Innovation Program of the Chinese Academy of Sciences (XDA16030301, YZ).

## Conflict of interest

The authors declare that the research was conducted in the absence of any commercial or financial relationships that could be construed as a potential conflict of interest.

## Publisher’s note

All claims expressed in this article are solely those of the authors and do not necessarily represent those of their affiliated organizations, or those of the publisher, the editors and the reviewers. Any product that may be evaluated in this article, or claim that may be made by its manufacturer, is not guaranteed or endorsed by the publisher.

## Glossary

**Table:**

Ank1	Ankyrin 1
Ap2a1	Adaptor related protein complex 2 subunit alpha 1
Ap2m1	Adaptor related protein complex 2 subunit mu 1
App	Amyloid beta precursor protein
Aqp1	Aquaporin 1
Arpc1b	Actin related protein 2/3 complex subunit 1B
Arpc2	Actin related protein 2/3 complex subunit 2
Arpc5	Actin related protein 2/3 complex subunit 5
Atp1a1	ATPase Na+/K+ transporting subunit alpha 1
Atp1b2	ATPase Na+/K+ transporting subunit beta 2
Atp4a	ATPase H+/K+ transporting subunit alpha
C/EBPs	CCAAT/enhancer-binding proteins
Cdh1	Cadherin 1
Cdkn2d	Cyclin dependent kinase inhibitor 2D
Cfd	Complement factor D
c-Fos	Fos proto-oncogene
c-Jun	Jun proto-oncogene
CMP	Common Myeloid Progenitors
Creb1	CAMP responsive element binding protein 1
Crebbp	CREB binding protein
Creld2	Cysteine rich with EGF like domains 2
Csf2rb	Colony stimulating factor 2 receptor beta common subunit
DEGs	Differentially Expressed Genes
Dmkn	Dermokine
EP300	E1A binding protein P300
Fcgr2b	Fc fragment of IgG receptor IIb
Fgl2	Fibrinogen like 2
FLT3	Fms related tyrosine kinase 3
Gabrr1	Gamma-aminobutyric acid type a receptor Rho1 subunit
GATA1	GATA-binding protein 1
GATA2	GATA-binding protein 2
Gfi1	Growth factor Independent 1 transcriptional repressor
Glt1d1	Glycosyltransferase 1 domain containing 1
GMP	Granulocyte monocyte progenitors
Gna14	G protein subunit alpha 14
Gnai3	G protein subunit alpha I3
Gnb4	G protein subunit beta 4
GO	Gene ontology
Gpc1	Glypican 1
GSEA	Gene set enrichment analysis
HMGN2	High mobility family nucleosome binding domain 2
HSCs	Hematopoietic stem cells
Hspa5	Heat shock protein family A (Hsp70) member 5
HSPC	Hematopoietic stem and progenitor cell
Ifnar	Interferon alpha and beta receptor subunit 1
Irf5	Interferon regulatory factor 5
Irf7	Interferon regulatory factor 7
IRF8	Interferon regulatory factor
KEGG	Kyoto encyclopedia of genes and genomes
Klf1	Kruppel like factor 1
KLF4	Kruppel-like factor 4
Lbp	Lipopolysaccharide binding protein
Lef1	Lymphoid enhancer binding factor 1
LMPs	Lymphoblastoid progenitor cells
Lyz	Lysozyme
Mef2a	Myocyte enhancer factor 2A
Nav2	Neuron navigator 2
Nfatc4	Nuclear factor of activated T cells 4
Nfia	Nuclear factor I A
NFkb1	Nuclear factor kappa b subunit 1
Oosp1	Oocyte secreted protein 1
Pglyrp1	Peptidoglycan recognition protein 1
Pi3k	Phosphatidylinositol-4,5-bisphosphate 3-kinase catalytic subunit delta
Pias1	Protein inhibitor of activated STAT 1
POMP	Proteasome maturation protein
Ppp3cb	Protein phosphatase 3 catalytic subunit beta
Prom1	Prominin 1
Ptk2b	Protein tyrosine kinase 2 beta
Rac	Rac Family small GTPase 1
Rnase10	Ribonuclease a family member 10
Rnase12	Ribonuclease a Family member 12
RNA-seq	RNA sequencing
Rtn4rl1	Reticulon 4 receptor like 1
Runx1	RUNX family transcription factor 1
Runx2	RUNX family transcription factor 2
Slc2a3	Solute carrier family 2 member 3
SPI1	Transcription factor PU.1
Srgn	Serglycin
Stat5b	Signal transducer and activator of transcription 5B
Stat6	Signal transducer and activator of transcription 6
TCA	Tricarboxylic acid cycle
Tnk2	Tyrosine kinase non-receptor 2
TPM	Transcripts per kilobase of exon model per million mapped reads
Tslp	Thymic stromal lymphopoietin
