# Supplementary material for: Central gene transcriptional regulatory networks shaping monocyte development in bone marrow
Source: Front Immunol. 2022 Oct 11;13:1011279. doi: 10.3389/fimmu.2022.1011279 (PMC9595600; doi:10.3389/fimmu.2022.1011279)
Supplement: Supplementary file 3 [file DataSheet_1.pdf]

Supplementary figures

Central gene transcriptional regulatory networks shaping monocyte development in bone marrow

Zhaoqi Zhang[1,2，*], Elhusseny A. Bossila[1,2,3,*], Ling Li[1,4], Songnian Hu[5,#], Yong Zhao[1,2,4,#]
[1] State Key Laboratory of Membrane Biology, Institute of Zoology, Chinese Academy of Sciences, Beijing China; [2] University of Chinese Academy of Sciences, Beijing China; [3] Biotechnology Department, Faculty of Agriculture Al-Azhar University, Cairo, Egypt; [4] Beijing Institute for Stem Cell and Regeneration, Beijing China [5] State Key Laboratory of Microbial Resources, Institute of Microbiology, Chinese Academy of Sciences, Beijing China.

Suppl. Fig. 1

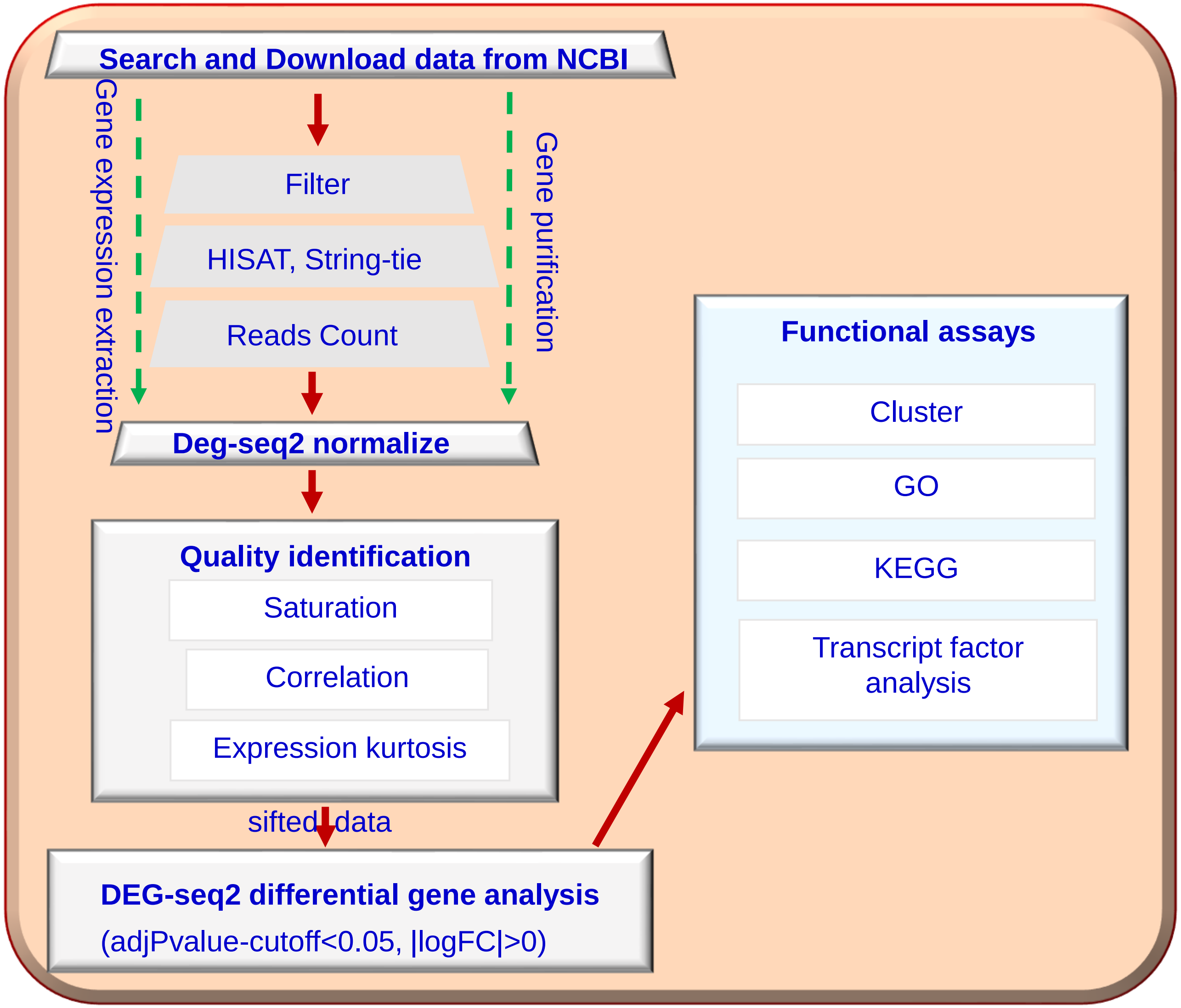

Supplementary figure 1. Data analysis process.
Our raw data and the raw data downloaded from NCBI were filtered and mapped to obtain gene expression and read count. Normalization and differential gene analysis were then performed using Deseq2 software, and the effects of differential genes in monocyte development were sought by various enrichment methods.

Suppl. Fig. 2

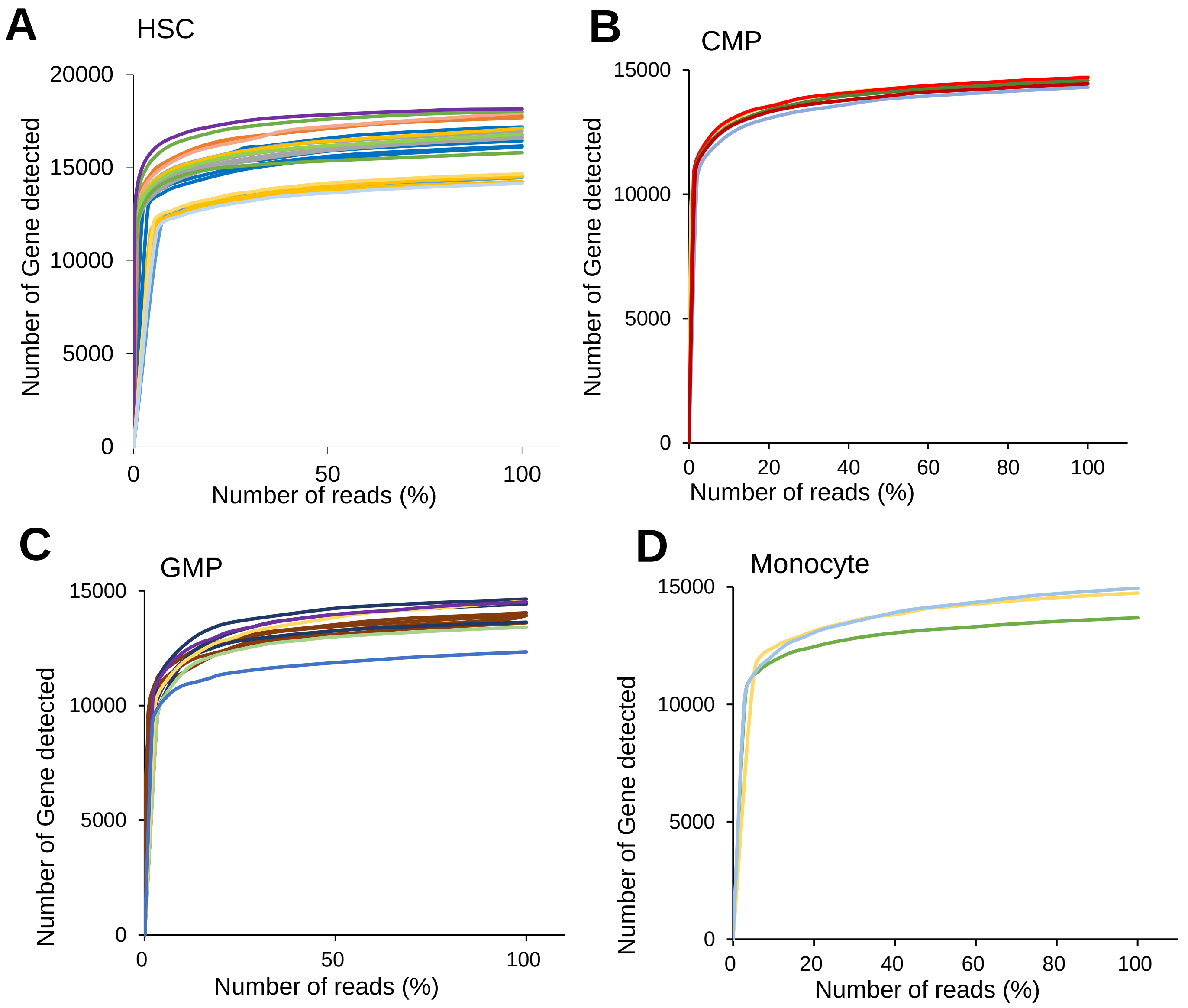

Supplementary figure 2. Data saturation analysis.
Saturation of each cell population, including HSC (A), CMP (B), GMP (C), and monocytes (D). The y-axis represents number of genes detected, and the x-axis represents the percentage of reads.

Suppl. Fig. 3

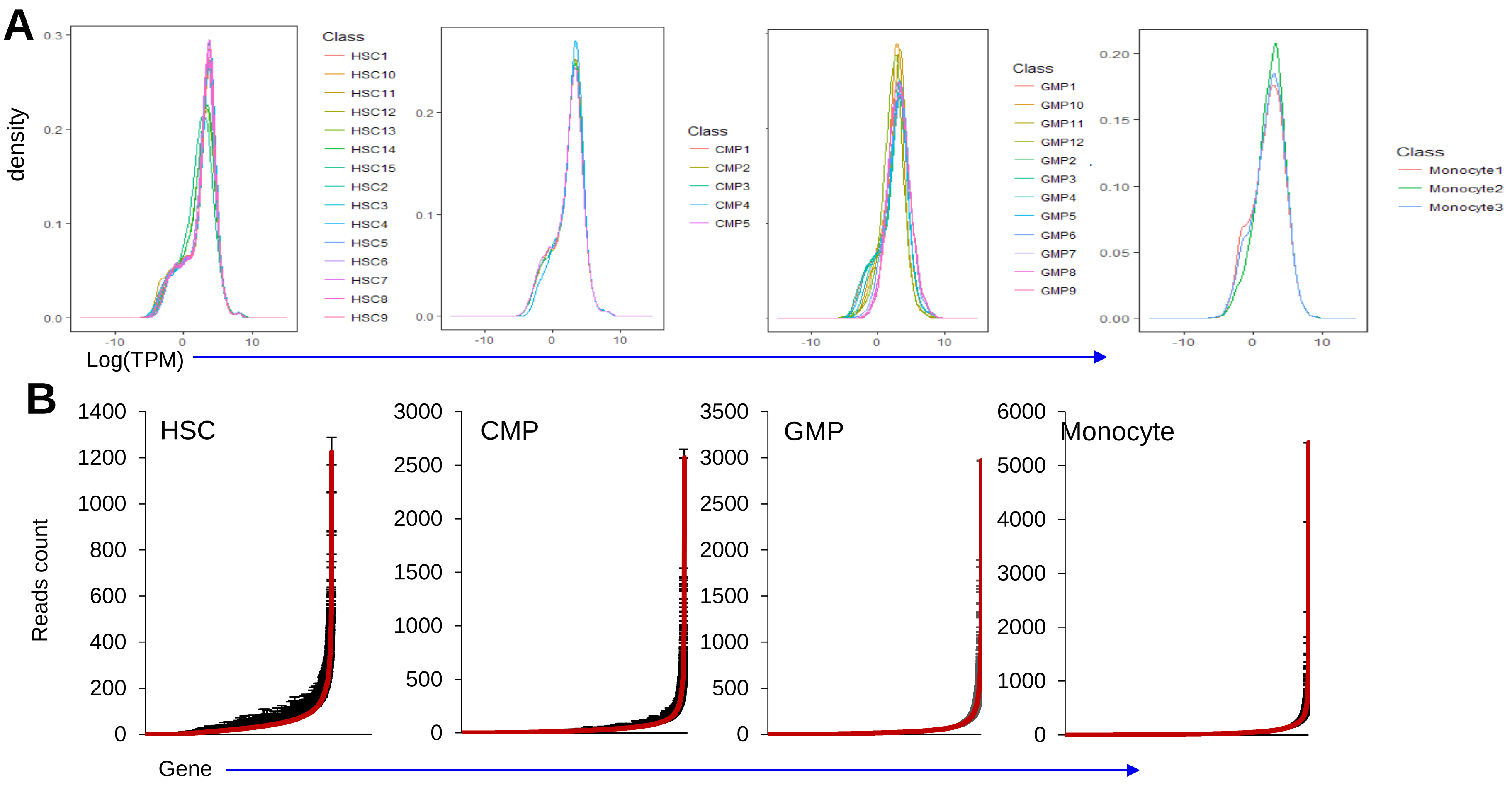

Supplementary figure 3. Data expression abundance analysis.

A. Density plots of log2-transformed TPM values. B. Gene expression and standard deviation curves

Suppl. Fig. 4

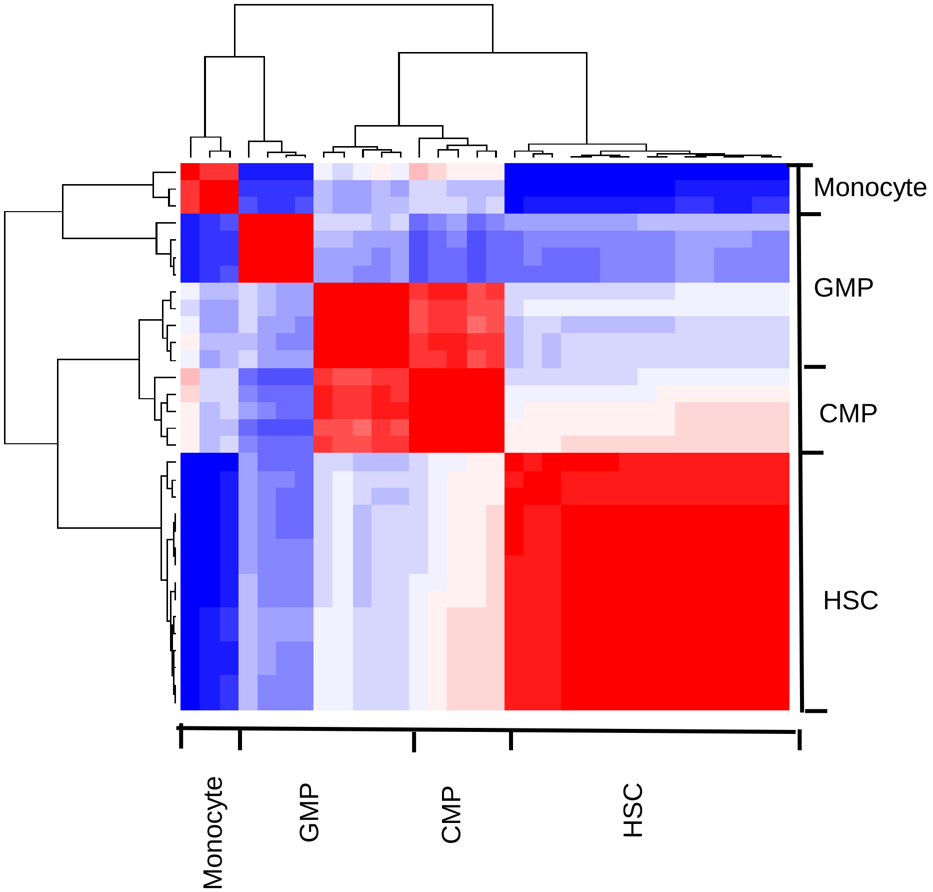

Supplementary figure 4. Corrplot of the download data.

Suppl. Fig. 5

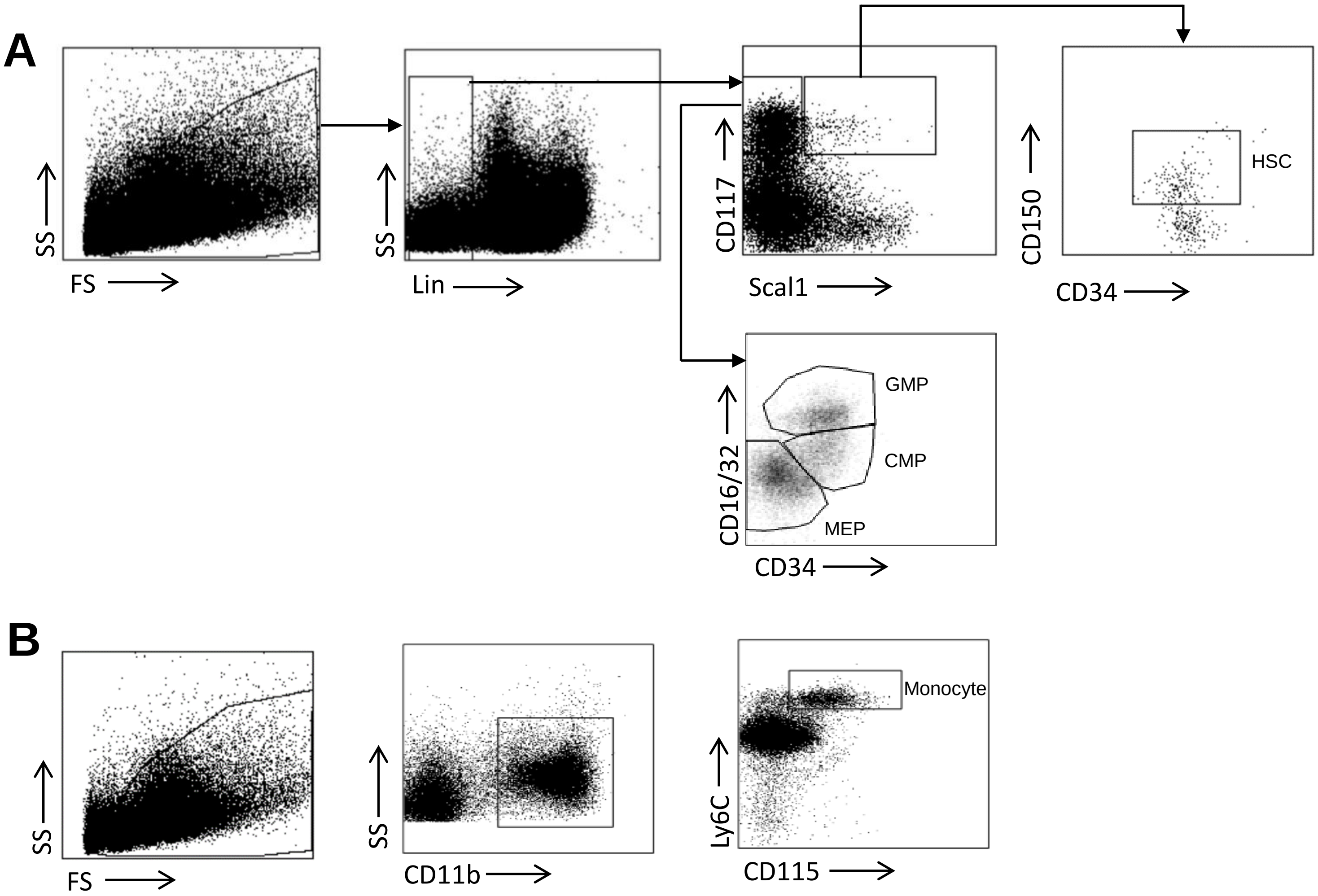

Supplementary figure 5. Extraction strategy for HSCs, CMPs, GMPs, and monocytes.
A. and B. Sorting strategies for mouse myeloid precursor cells (HSCs, CMPs, and GMPs) and monocytes, respectively..

Suppl. Fig. 6

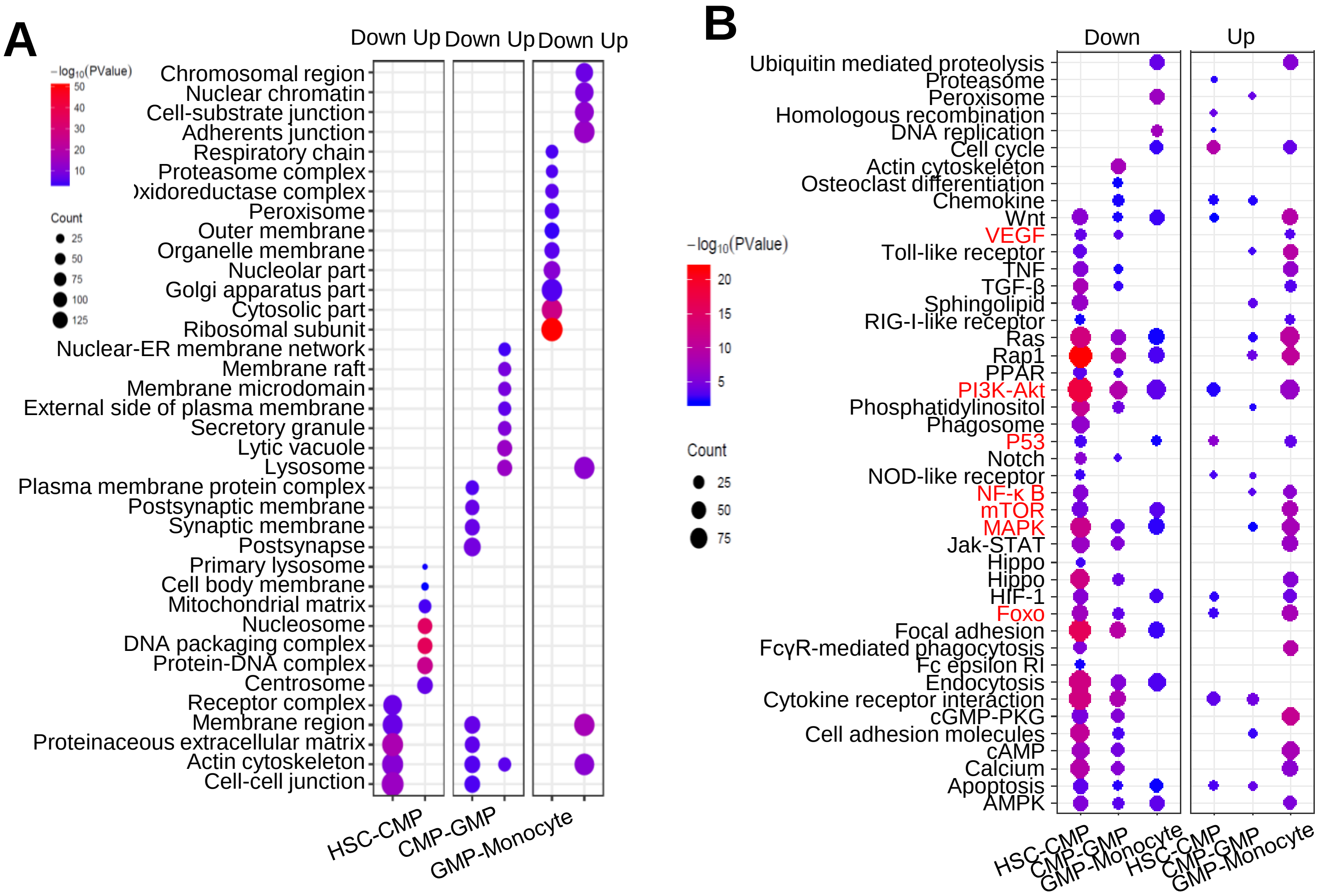

Supplementary figure 6. KEGG and GO enrichment of DEGs during monocyte development.
A. The bubble plot of cell component changes during monocyte development. The size of the bubble indicates different cell components' gene count. B. the signaling pathway changes during mouse monocyte development. The size of the bubble indicates the gene counts of different pathways.

Suppl. Fig. 7

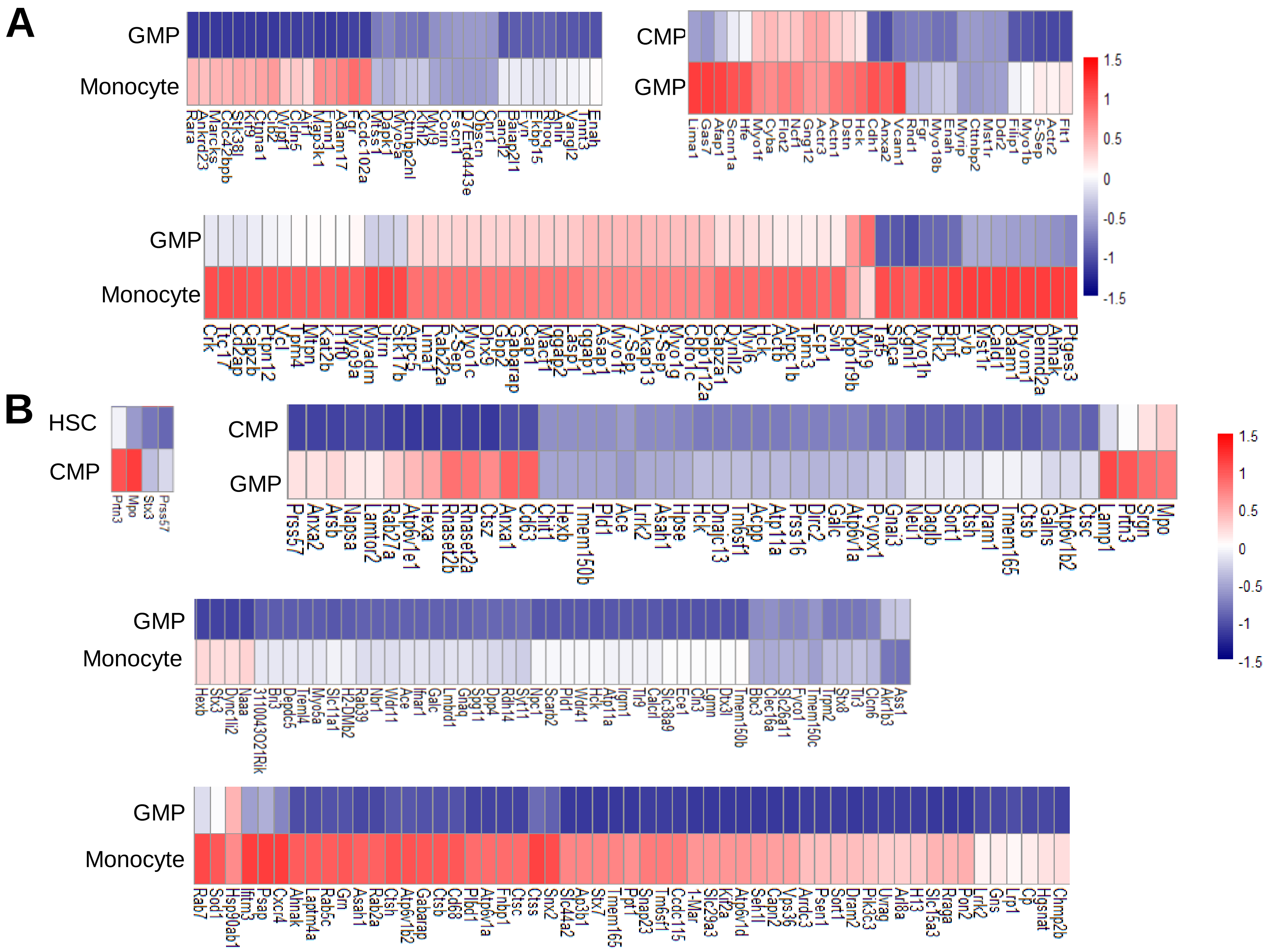

Supplementary figure 7. Changes in main cell localization regulate the development of monocytes.

A. Heatmap of the genes associated with actin cytoskeleton and membrane region during monocyte development.

B. Heatmap of the upregulated genes associated with lysosomes during monocyte development stages.

Suppl. Fig. 8

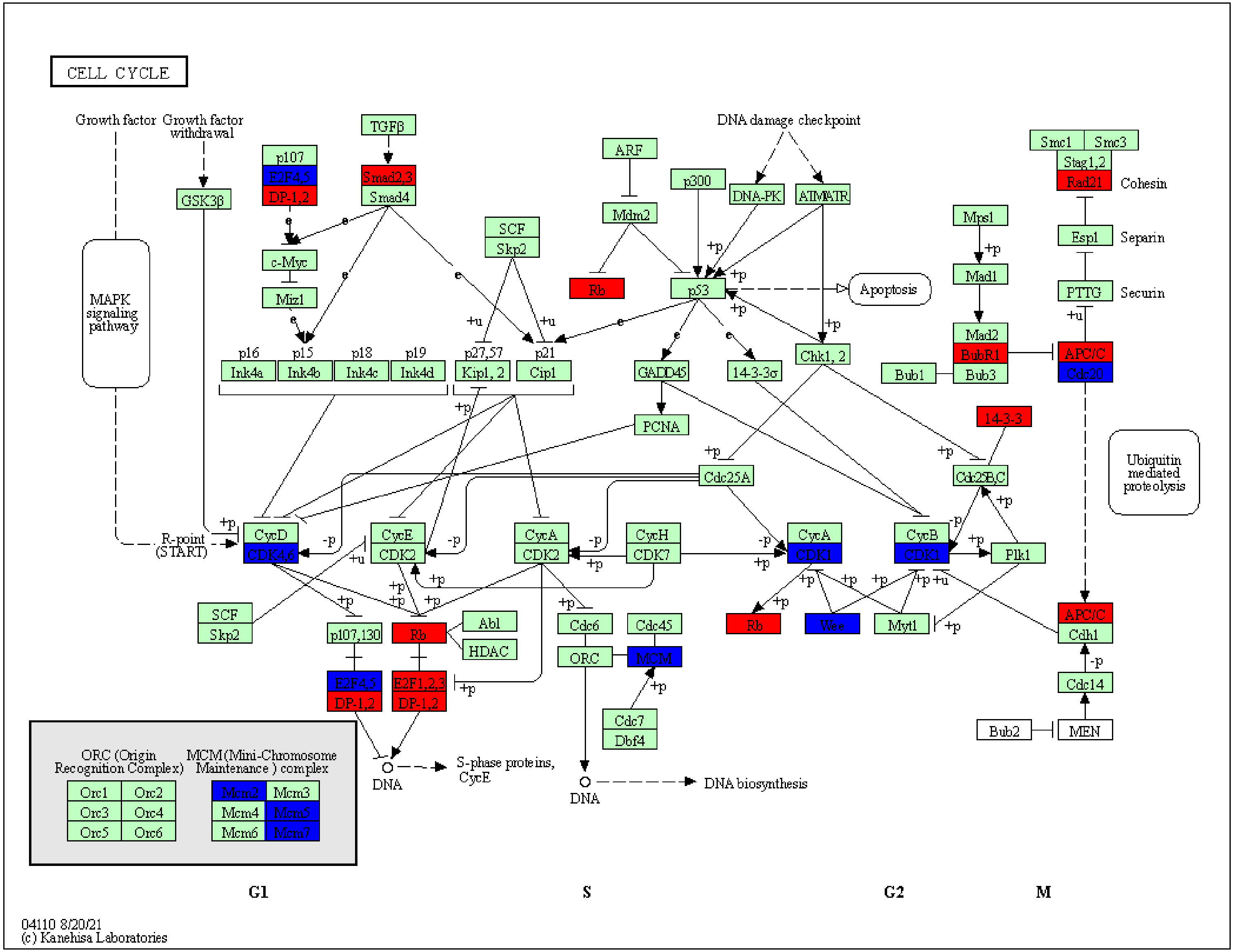

Supplementary figure 8. cell cycle changes in cluster3 and cluster6.

Suppl. Fig. 9

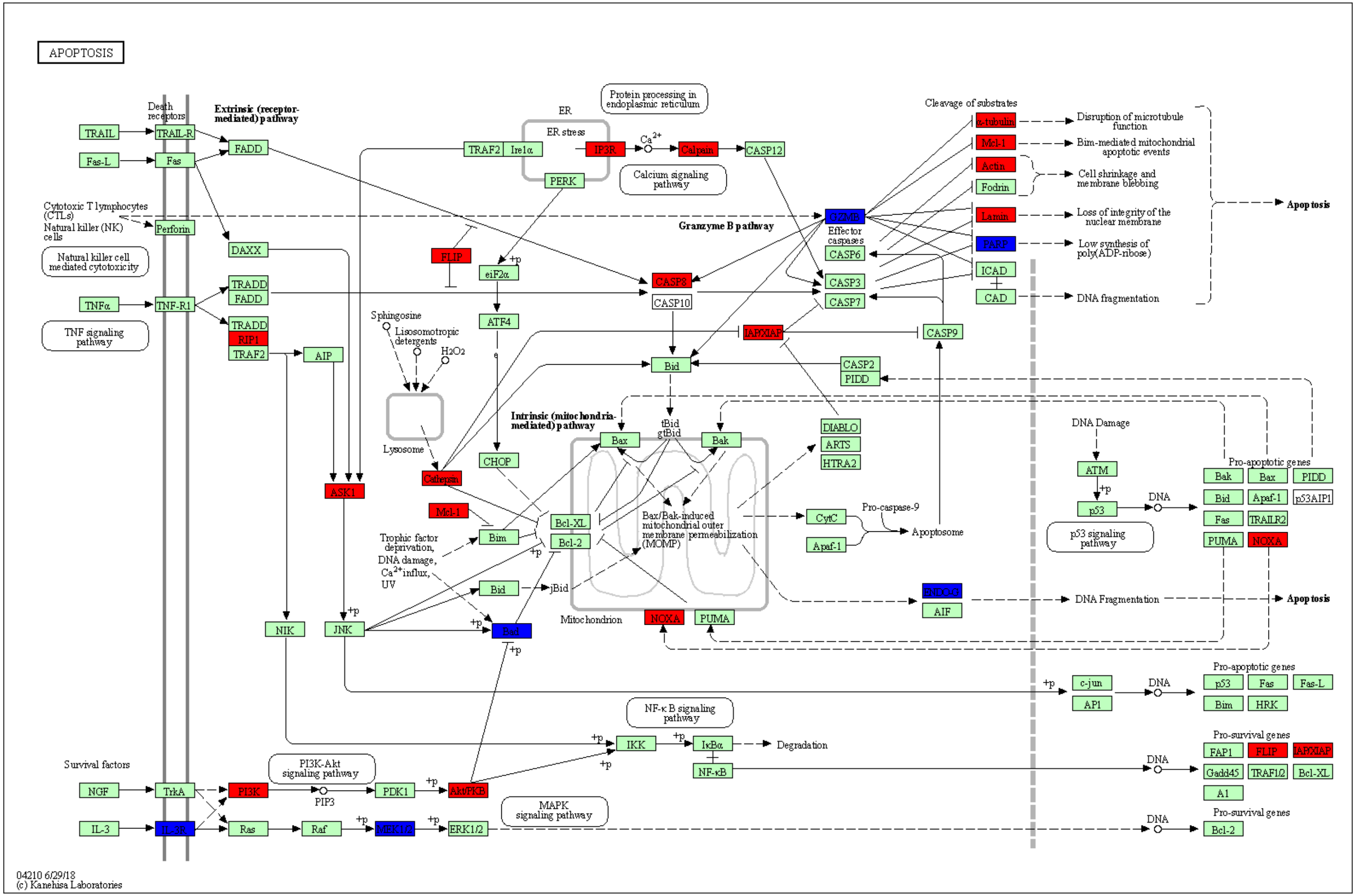

Supplementary figure 9. Apoptosis changes in cluster3 and cluster6.

Suppl. Fig. 10

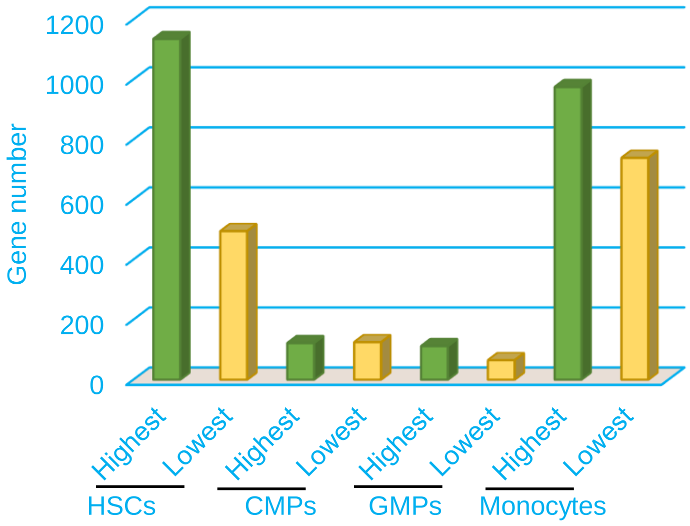

Supplementary figure 10. Specific high and low expression genes at various stages of monocyte development (|logFC|>0, adjqvalue<0.05).

Suppl. Fig. 11

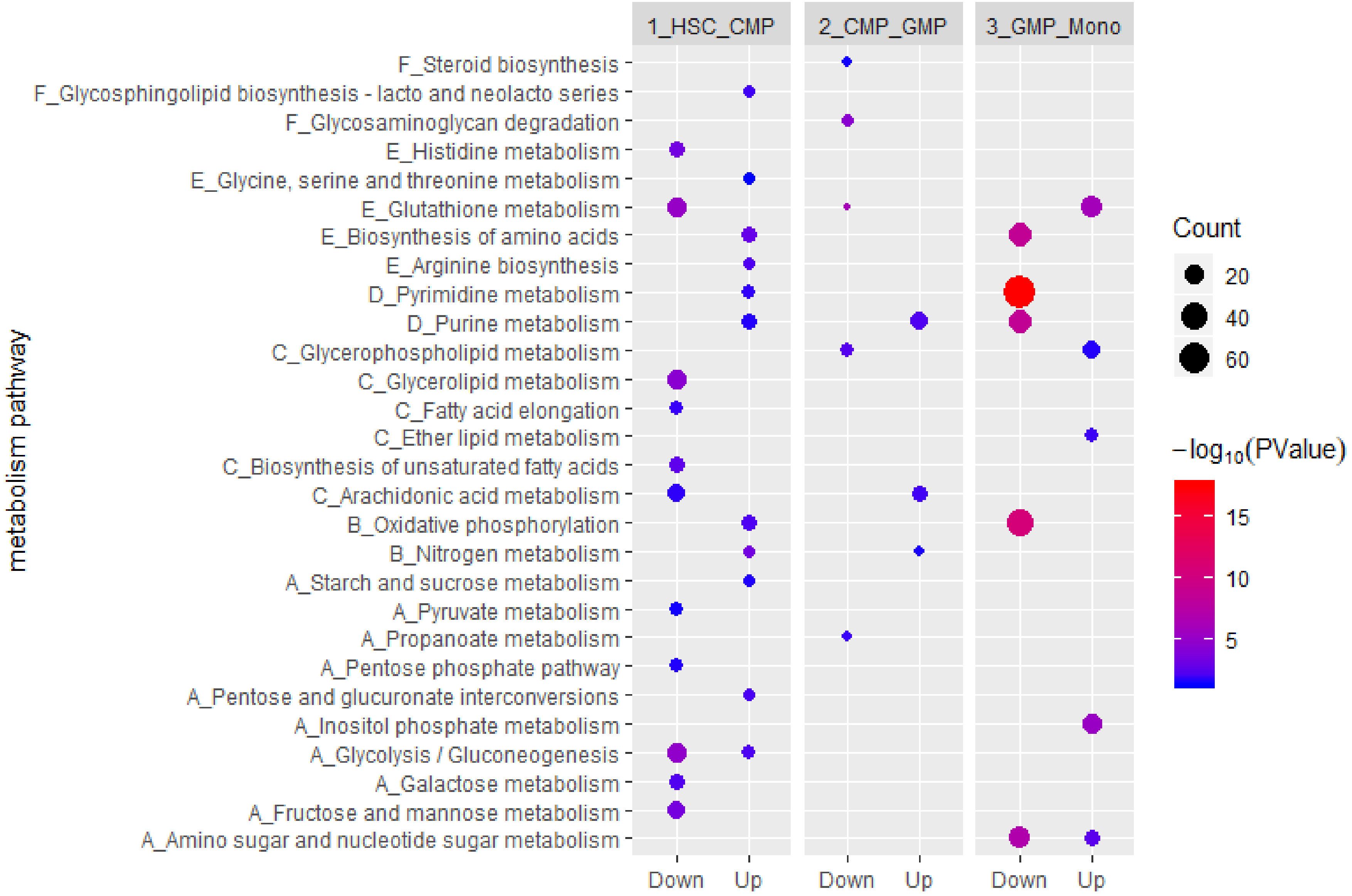

Supplementary figure 11. Metabolism changes during mouse monocyte development stages.
The bubble plot of cell metabolism changes during monocyte development. The size of the bubble indicates different cell components' gene count. The color of the bubble indicates a different -log10(Pvalue).

Suppl. Fig. 12

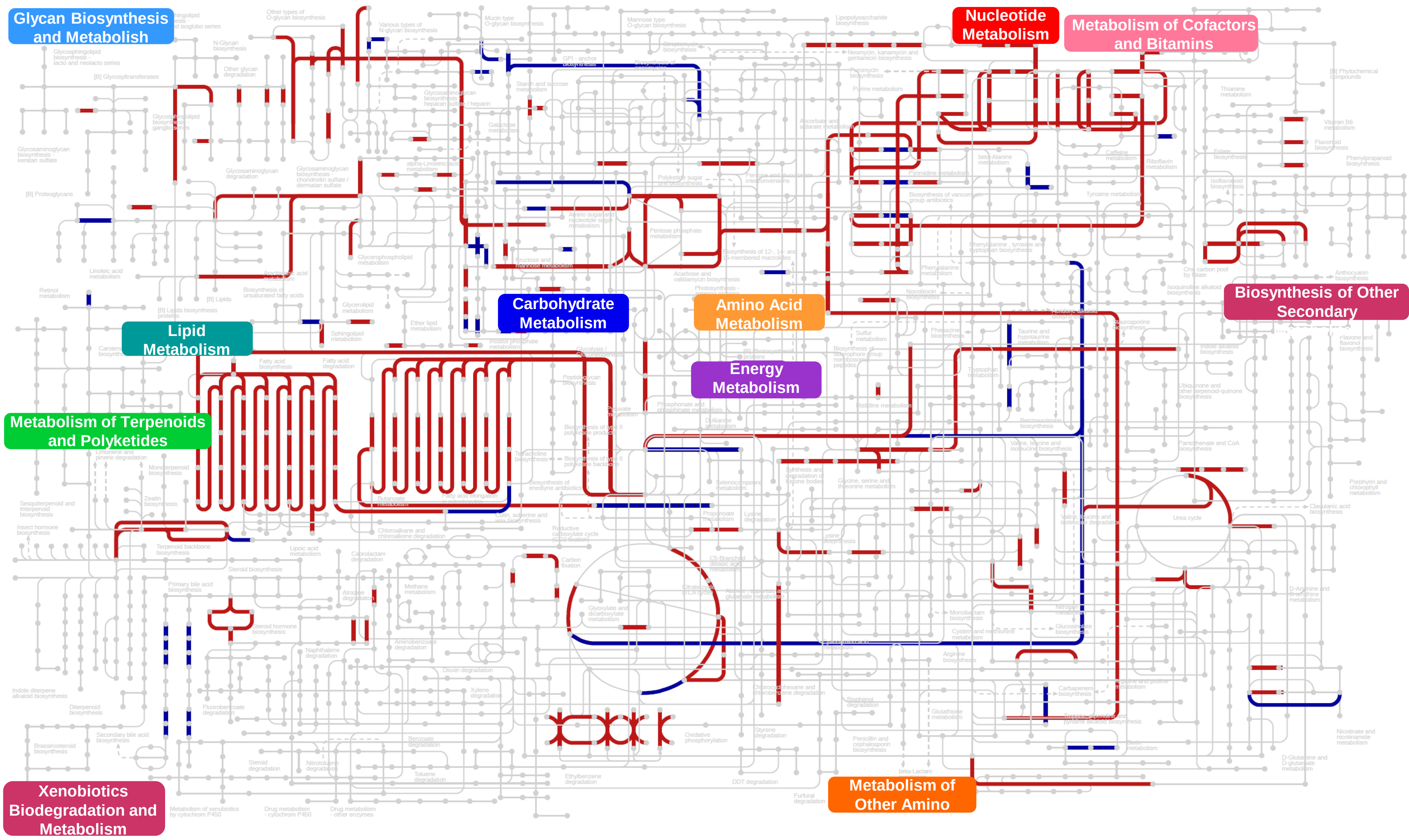

Supplementary figure 12. Metabolism changes in CMPs to GMPs.
Metabolism network of the genes transcriptionally regulated during the differentiation of CMPs into GMPs.

Suppl. Fig. 13

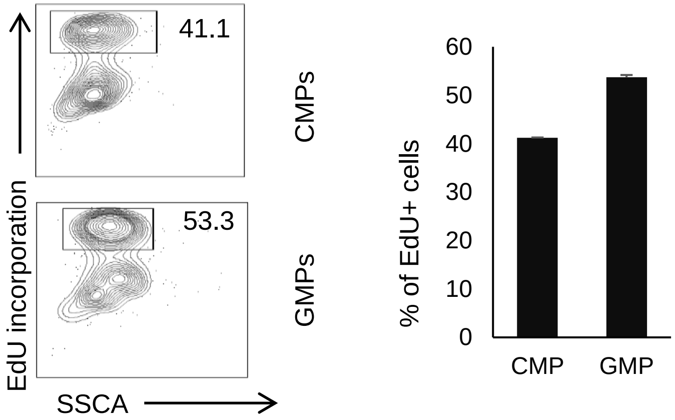

Supplementary figure 13. FCS and histogram results of EdU incorporation in CMPs and GMPs.

Suppl. Fig. 14

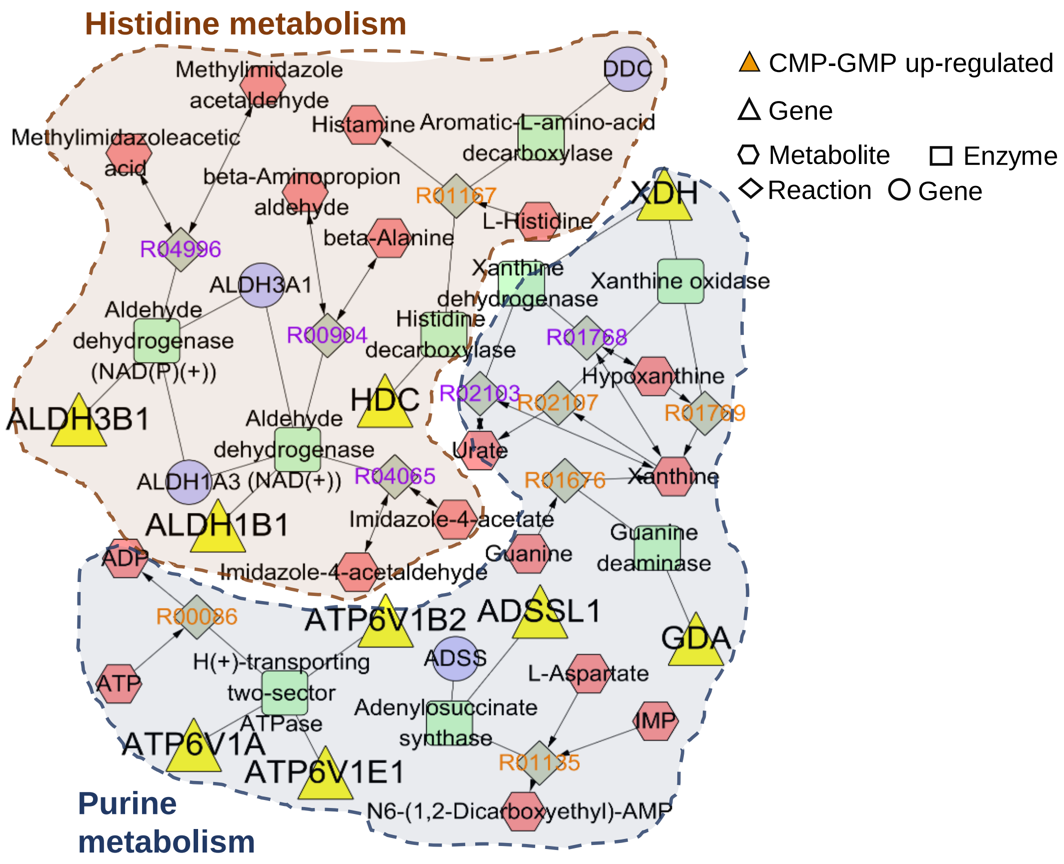

Supplementary figure 14. Nucleic acid metabolism changes in CMPs to GMPs.
Upregulated genes' Metscape metabolism network of histidine metabolism pathway and purine metabolism pathway in differentiation of mouse CMPs into GMPs.

Suppl. Fig. 15

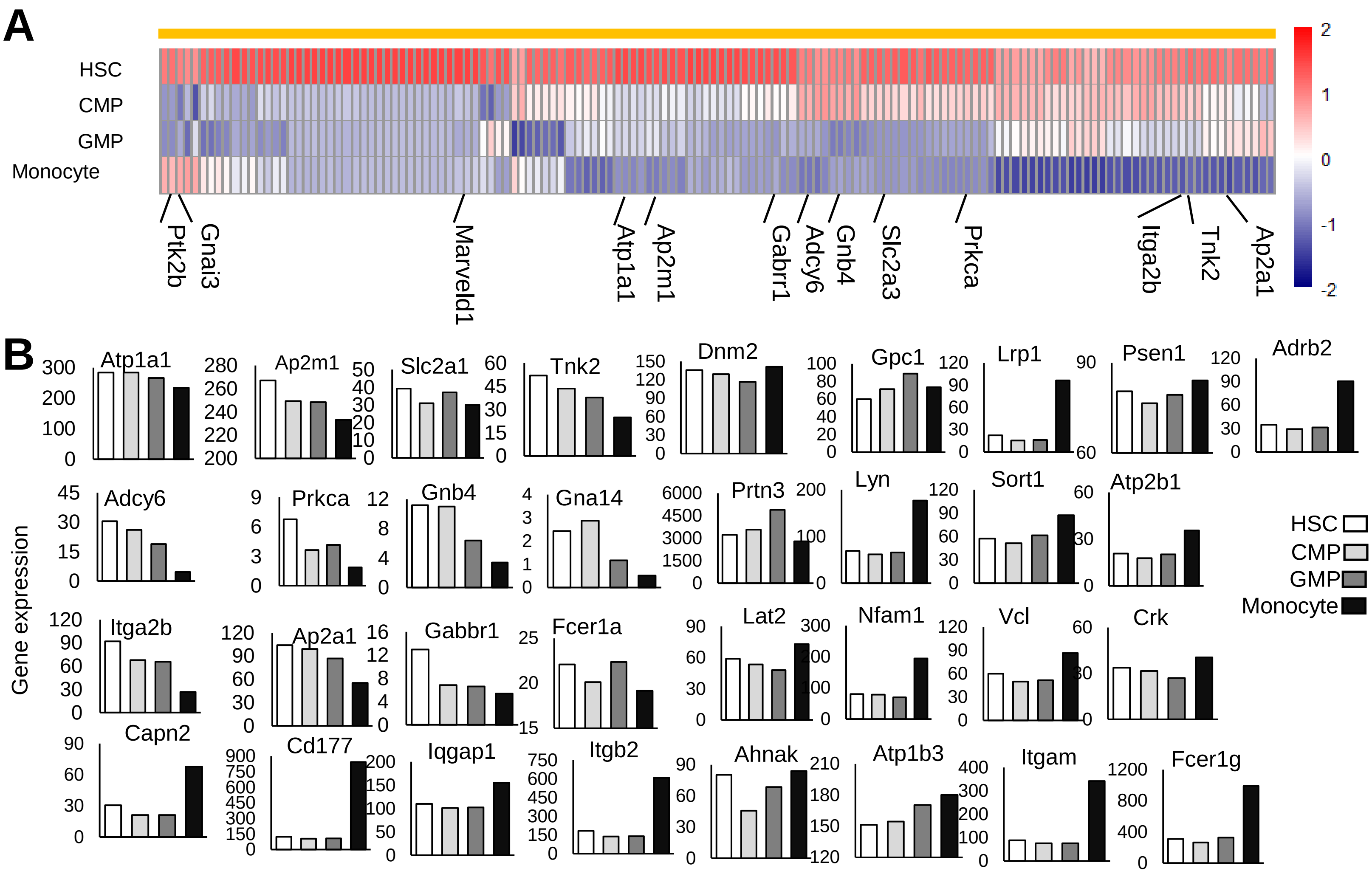

Supplementary figure 15. Specific high and low expression genes at various stages of mouse monocyte development.
A. Heatmap of highly expressed membrane molecular genes in HSCs. B. RNA-seq results of the sorted cells by our laboratory.

Suppl. Fig. 16

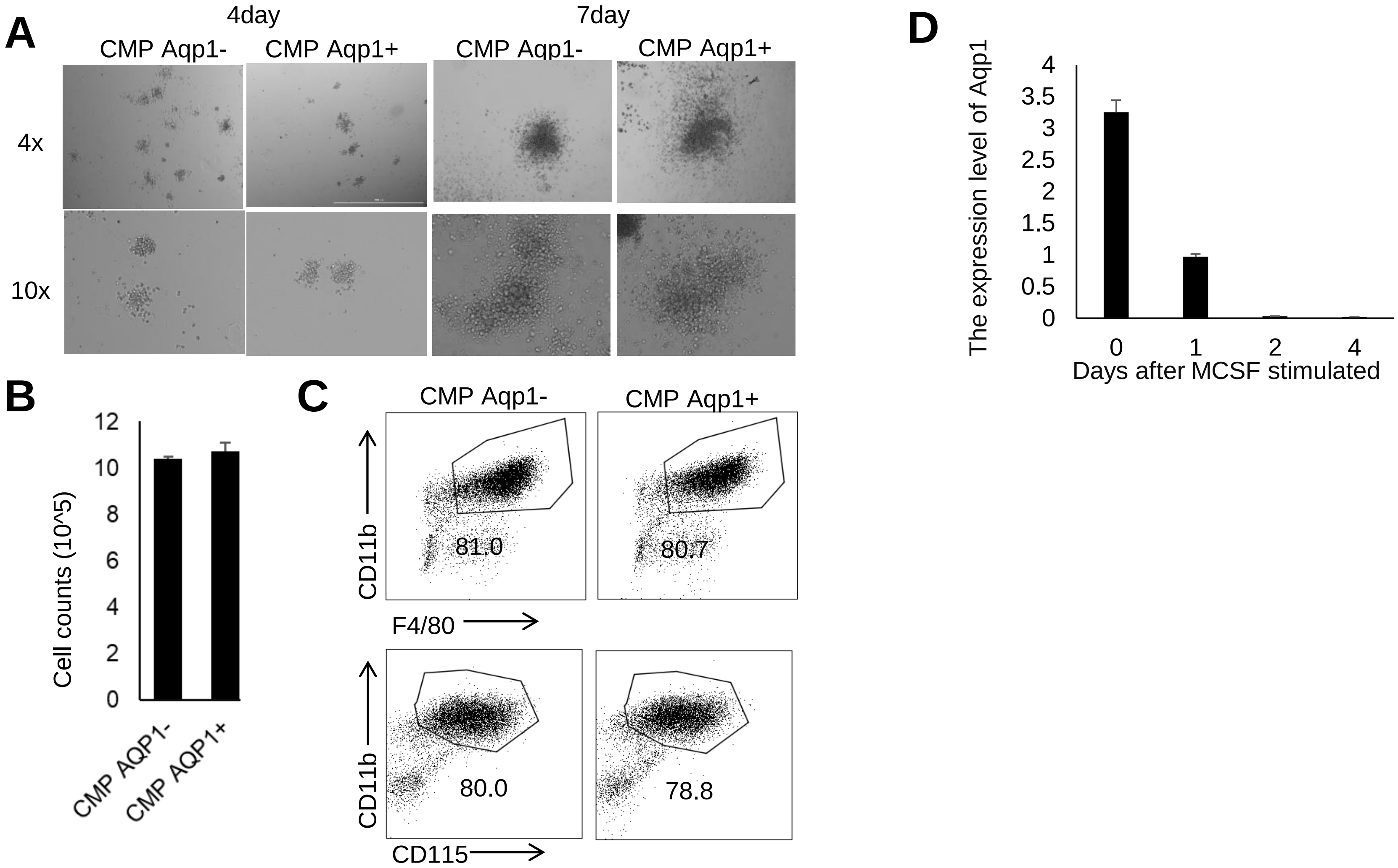

Supplementary figure 16. The expression of Aqp1 in CMP had no effect on the differentiation function of CMP. We sorted Aqp1+CMPs and Aqp1-CMPs for colony formation with SCF: 50ng/ml, IL3: 10ng/ml, IL-6: 20ng/ml, M-CSF: 20ng/ml. A, B and C. photographs, cell counts and FCS results of Aqp1+CMPs and Aqp1-CMPs colony formation result. Aqp1+CMPs were sorted and treated with 20ng/ml MCSF for 0,1,2,4 days, and the expression of Aqp1 was shown in D.

Suppl. Fig.17

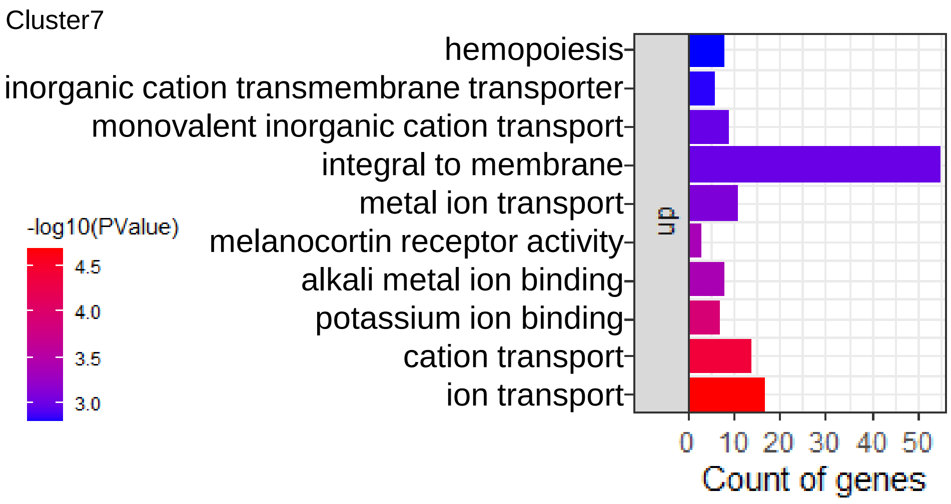

Supplementary figure 17. KEGG enrichment of genes in cluster7.
Color represents the -log10(Pvalue), with red representing high significances and blue indicating lower significances.

Suppl. Fig. 18

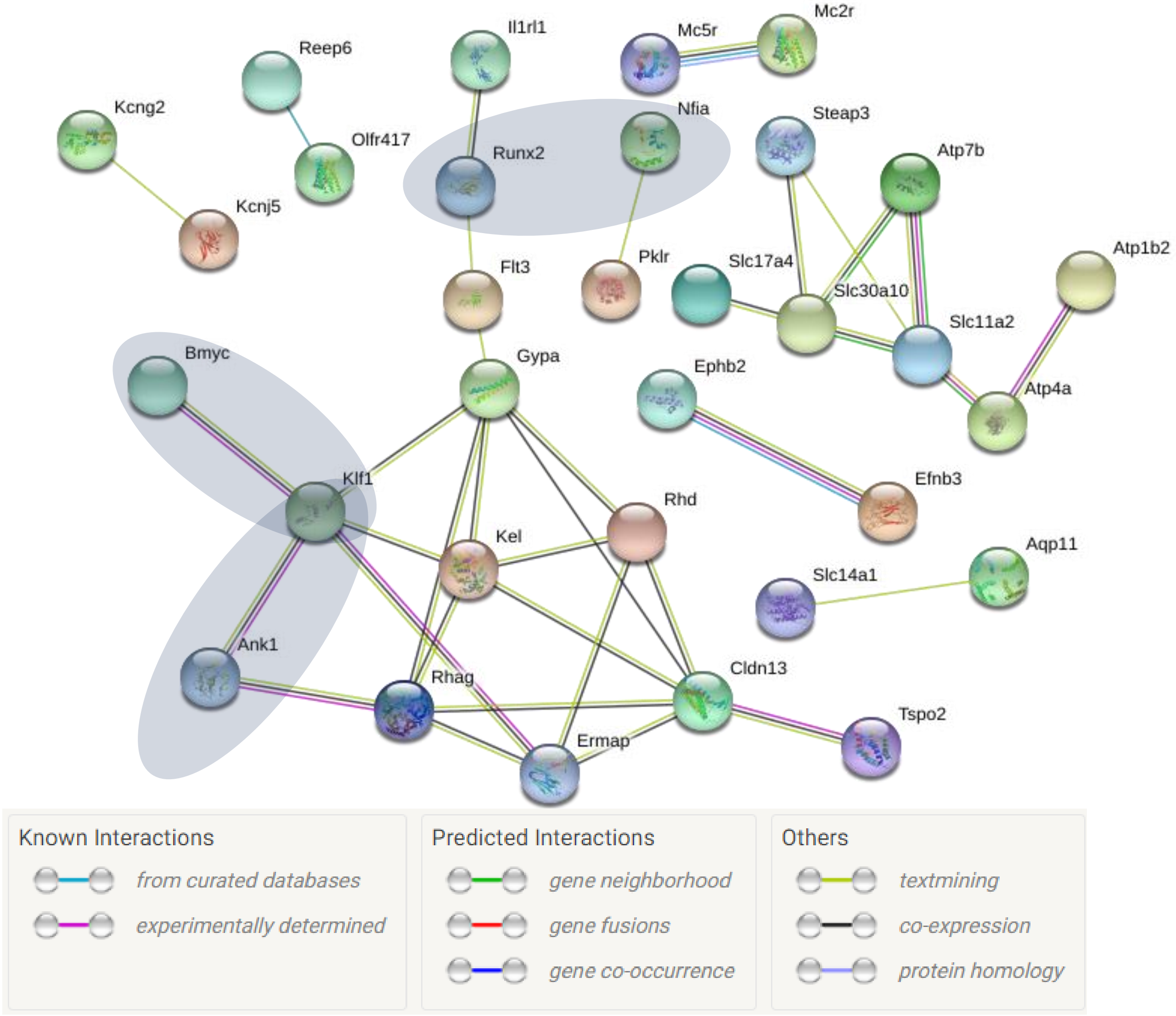

Supplementary figure 18. Transcript factor regulation of ion transport network in the CMP stage. The selected genes are the transcription factors upregulated in CMPs.

Suppl. Fig. 19

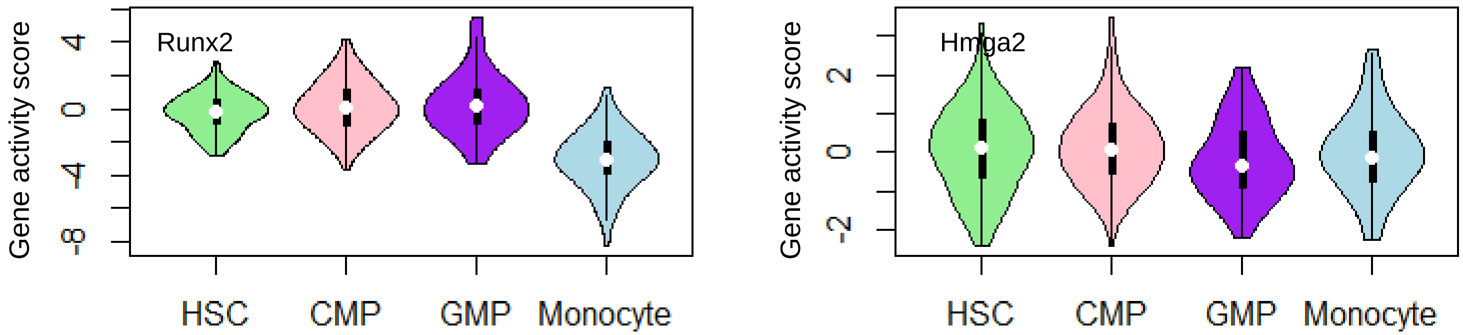

Supplementary figure 19. Violin plot of Transcript factors regulation trend.

Suppl. Fig. 20

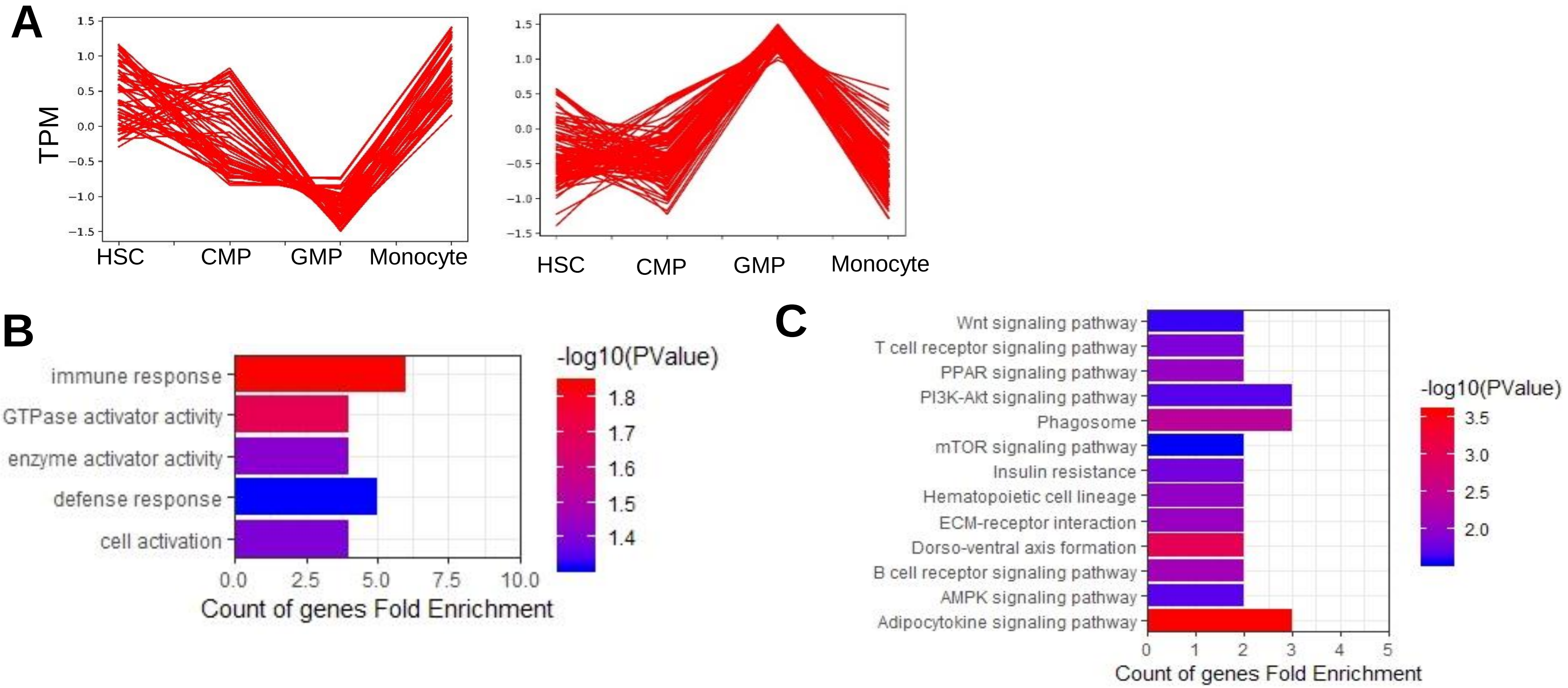

Supplementary figure 20. Enrichment of significantly downregulated genes differentiate genes in mouse GMPs in cluster.
A. Line chart of the genes TPM regulation mode of significantly downregulated genes during the mouse GMP stage. B. and C. GO enrichment and KEGG pathway enrichment of significantly downregulated genes in mouse GMPs.

Suppl. Fig. 21

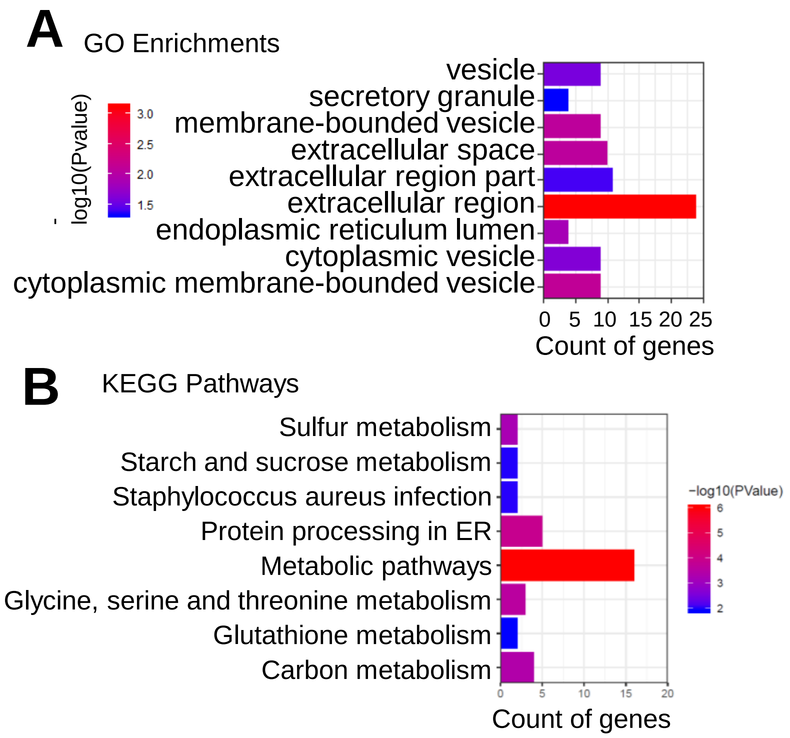

Supplementary figure 21. Enrichment of significantly upregulated genes differentiate genes in mouse GMPs in cluster.

A. Histogram of the GO pathways of highly expressed genes in GMPs. **B.** KEGG enrichment of highly expressed genes in GMPs

Suppl. Fig. 22

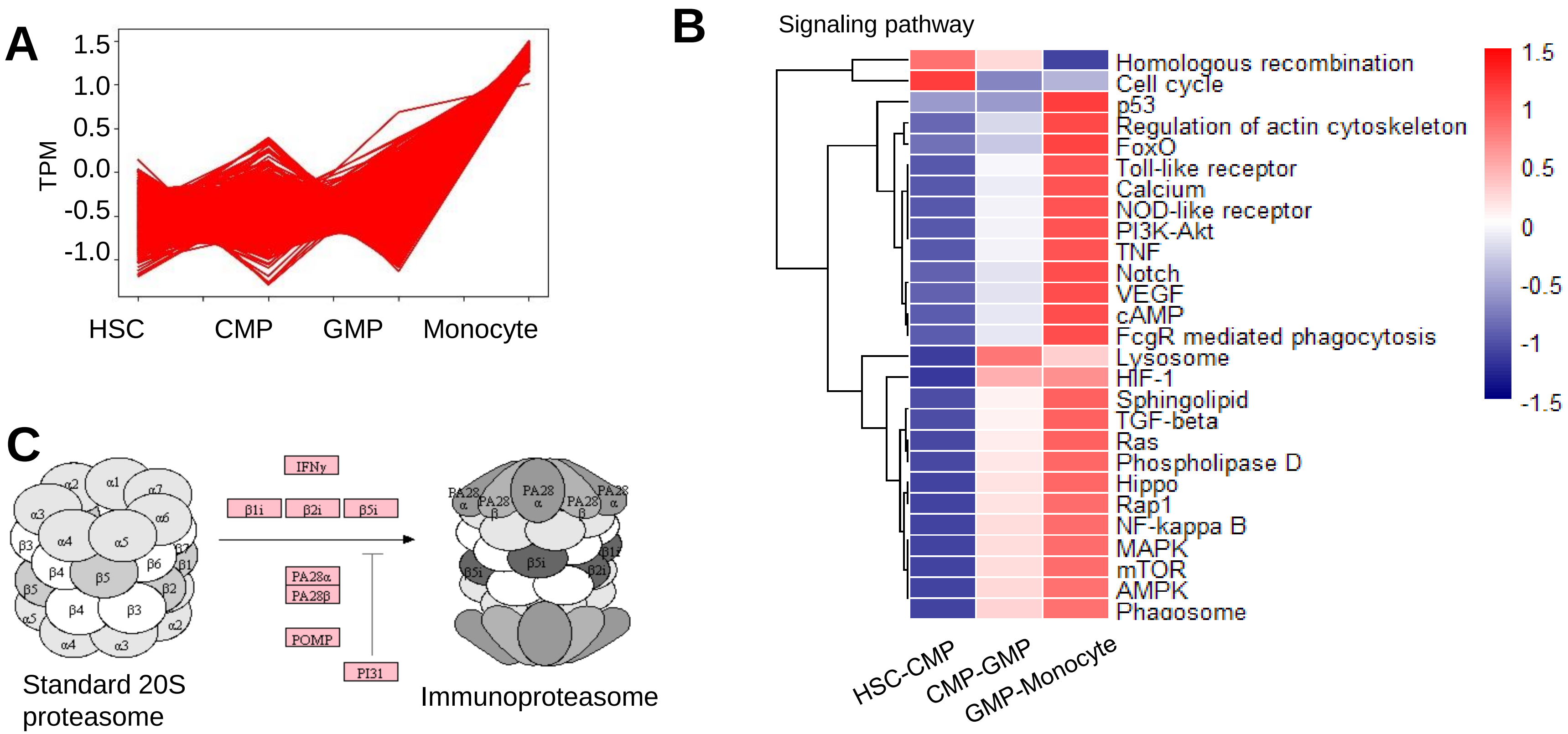

Supplementary figure 22. Enrichment of significantly downregulated genes differentiate genes in mouse GMPs in cluster.

A. Line chart of the genes TPM regulation mode of significantly downregulated genes during the mouse GMP stage. B. and C. GO enrichment and KEGG pathway enrichment of significantly downregulated genes in mouse GMPs.

Suppl. Fig. 23

A

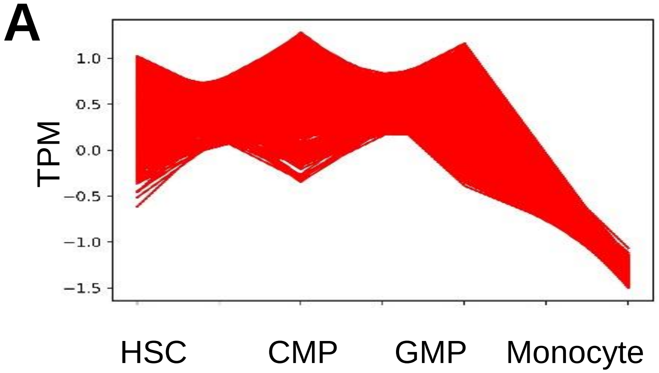

B

#Term	ID	Input number	Background number	P-Value
Ribosome	mmu03010	49	175	1.61E-37
Metabolic pathways	mmu01100	109	1494	1.00E-31
Huntington disease	mmu05016	30	194	4.14E-17
Oxidative phosphorylation	mmu00190	26	134	4.17E-17
Parkinson disease	mmu05012	26	144	1.94E-16
Alzheimer disease	mmu05010	26	175	1.21E-14
Thermogenesis	mmu04714	29	232	2.03E-14
DNA replication	mmu03030	13	35	3.24E-12
RNA transport	mmu03013	21	167	7.24E-11
Nucleotide excision repair	mmu03420	11	43	4.17E-09
Pyrimidine metabolism	mmu00240	12	58	6.58E-09
Non-alcoholic fatty liver disease (NAFLD)	mmu04932	17	151	2.07E-08
Mismatch repair	mmu03430	7	22	8.46E-07
Biosynthesis of amino acids	mmu01230	11	78	8.80E-07
Carbon metabolism	mmu01200	13	121	1.53E-06
RNA polymerase	mmu03020	7	30	4.90E-06
Antifolate resistance	mmu01523	7	30	4.90E-06
Purine metabolism	mmu00230	13	136	5.00E-06
One carbon pool by folate	mmu00670	6	19	5.47E-06
Ribosome biogenesis in eukaryotes	mmu03008	12	116	5.48E-06

Supplementary figure 23. Downregulated genes in monocytes.
A. Line chart of the genes TPM regulation mode of significantly downregulated genes during the monocyte stage. B. Summary of KEGG enrichment of downregulated genes in monocytes.

Suppl. Fig. 24

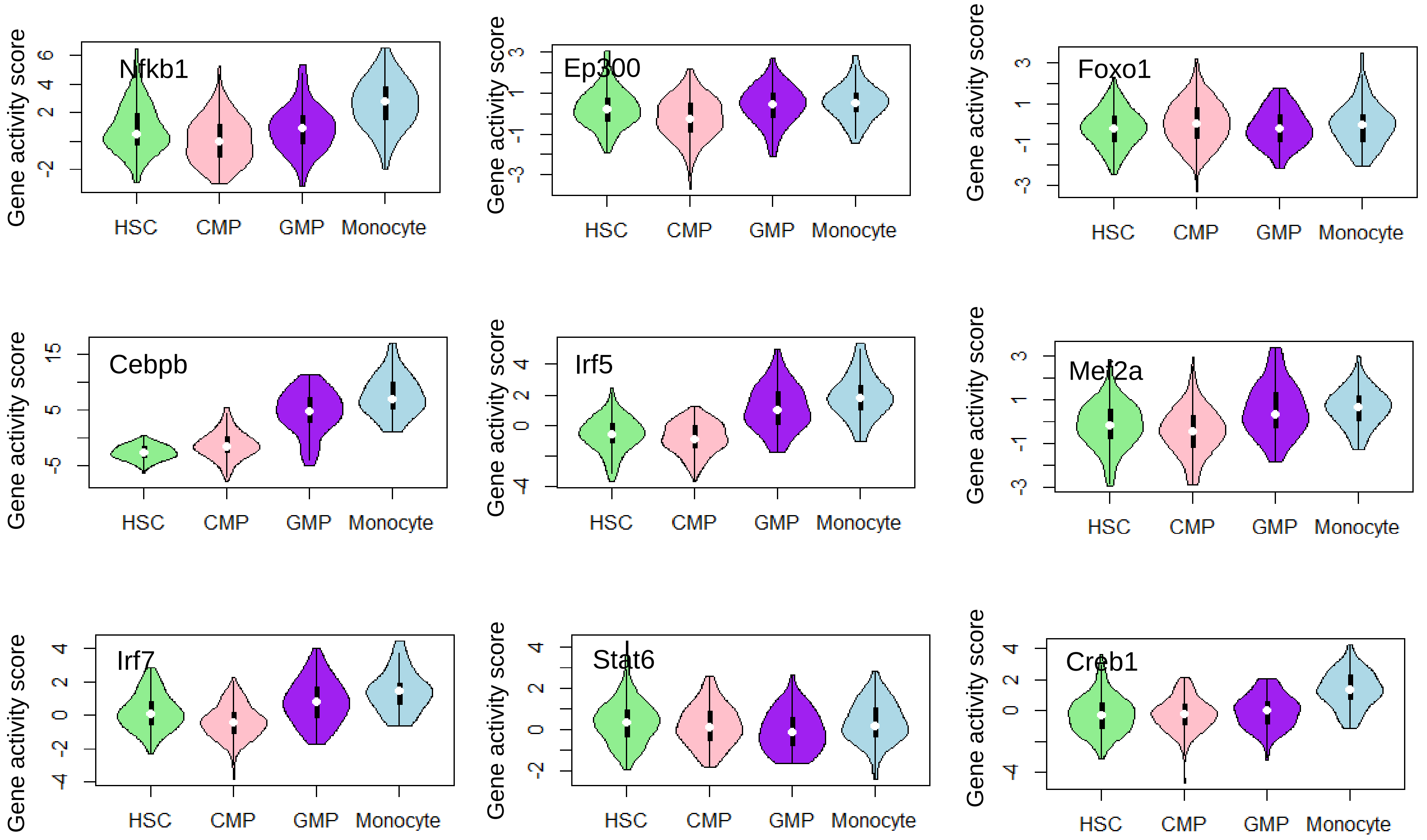

Supplementary figure 24. Violin plot of Transcripe factors regulation trend

Suppl. Fig. 25

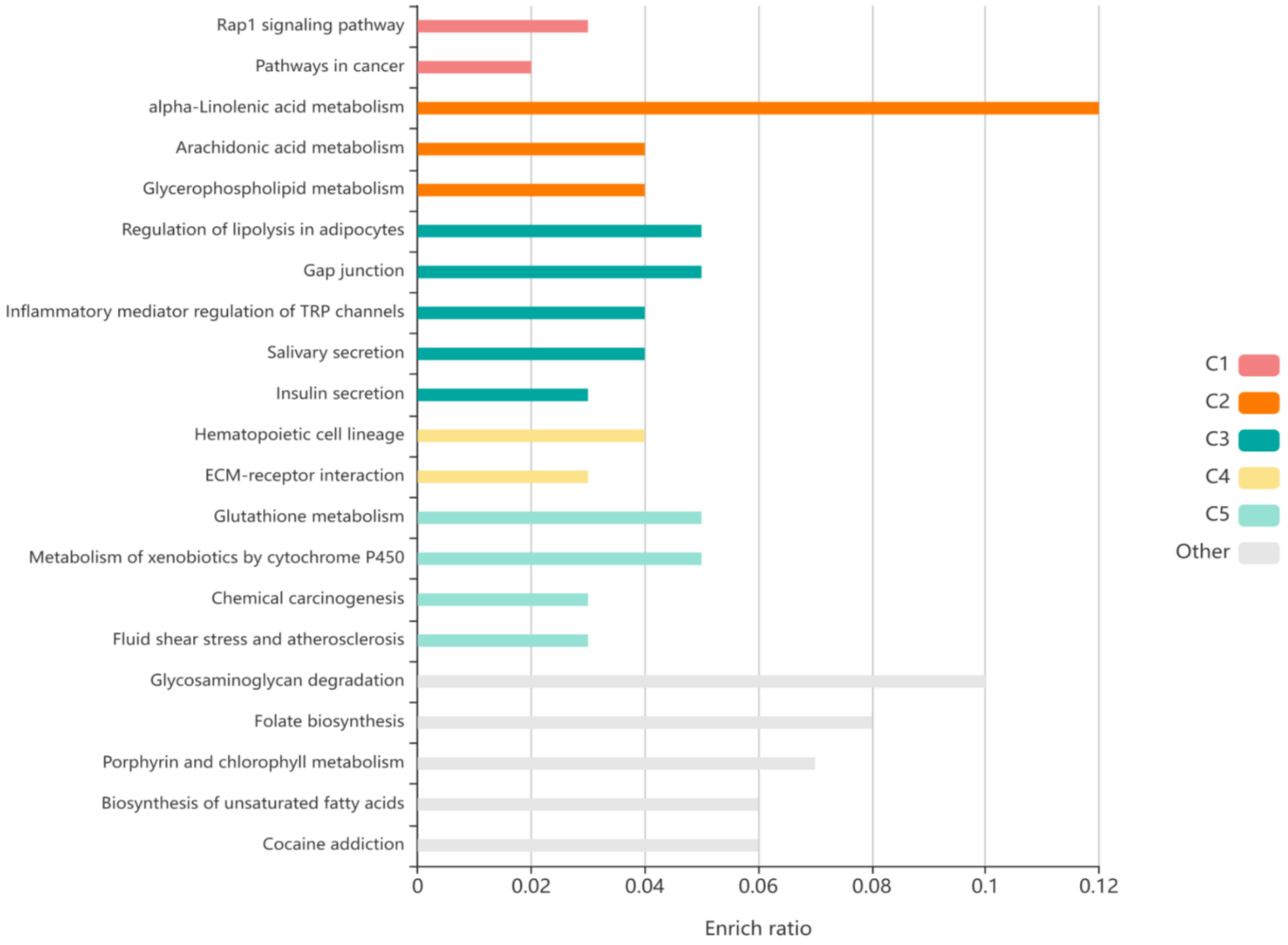

Supplementary figure 25. KEGG enrichment histogram of genes consistently downregulated during monocyte development.

The color represents different clusters.
