# Supplementary material for: Central gene transcriptional regulatory networks shaping monocyte development in bone marrow
Source: Front Immunol. 2022 Oct 11;13:1011279. doi: 10.3389/fimmu.2022.1011279 (PMC9595600; doi:10.3389/fimmu.2022.1011279)
Supplement: Supplementary file 4 [file DataSheet_2.docx]

**Suppl. Table1. Information of the download data**

| **Cell_Type** | **No.** | **Author** | **Journal** | **Title** | **PMID** |
| --- | --- | --- | --- | --- | --- |
| HSC | 1 | Jeong, M. et al. | Nat Genet | Large conserved domains of low DNA methylation maintained by Dnmt3a | 24270360 |
|  | 2 | Sun, D. et al. | Cell Stem Cell | Epigenomic profiling of young and aged HSCs reveals concerted changes during aging that reinforce self-renewal | 24792119 |
|  | 3 | Taiwo, O. et al. | Epigenetics | DNA methylation analysis of murine hematopoietic side population cells during aging | 23949429 |
| CMP | 1 | George. et al. | Nat Commun. | Next Generation Sequencing profile of leukemia from distinct cells-of-origin | 27397025 |
| GMP | 1 | George J, et al. | Nat Commun. | Next Generation Sequencing profile of leukemia from distinct cells-of-origin | 27397025 |
|  | 2 | Stavropoulou V, et al. | Cancer Cell. | Conditional modeling of MLL-AF9 induced leukemia revealed a stem cell derived aggressive disease | 27344946 |
|  | 3 | Shih AH, et al. | Cancer Cell. | Tet2-/-Flt3ITD and WT stem and progenitor cells | 25873173 |
| Monocyte | 1 | Pong WW, et al. | PLoS One | F11R is a novel monocyte prognostic biomarker for malignant glioma | 24147027 |

**Suppl. Table 2.** **The RNA-seq Data of HSC, CMP, GMP, and monocyte in different laboratories**

| **Cell_Type** | **No.** | **Platform** | **Mouse Strain** | **Mouse Age** | **Mouse Genotype** | **Cell Resource** | **Cell surface marker** | **Sample NO.** | **SRX** | **LibraryLayout** | **Platform** |
| --- | --- | --- | --- | --- | --- | --- | --- | --- | --- | --- | --- |
| HSC | 1 | Illumina HiSeq 2000 | C57BL/6 | 12 months | wild type | Bone marrow | SP-KSL-CD150+ | HSC_1 | SRX333409 | PAIRED | ILLUMINA |
|  |  |  |  |  |  |  |  | HSC_2 | SRX333408 | PAIRED | ILLUMINA |
|  |  |  |  |  |  |  |  | HSC_3 | SRX333407 | PAIRED | ILLUMINA |
|  |  |  |  |  |  |  |  | HSC_4 | SRX333406 | PAIRED | ILLUMINA |
|  | 2 | Illumina HiSeq 2000 | C57BL/6 | 24 months | WT | Bone marrow | SP-KSL-CD150+ | HSC_5 | SRX299570 | PAIRED | ILLUMINA |
|  |  |  |  |  |  |  |  | HSC_6 | SRX299569 | PAIRED | ILLUMINA |
|  |  |  |  |  |  |  |  | HSC_7 | SRX299568 | PAIRED | ILLUMINA |
|  |  |  |  |  |  |  |  | HSC_8 | SRX299567 | PAIRED | ILLUMINA |
|  |  |  |  | 4 months |  |  |  | HSC_9 | SRX299566 | PAIRED | ILLUMINA |
|  |  |  |  |  |  |  |  | HSC_10 | SRX299565 | PAIRED | ILLUMINA |
|  |  |  |  |  |  |  |  | HSC_11 | SRX299564 | PAIRED | ILLUMINA |
|  |  |  |  |  |  |  |  | HSC_12 | SRX299563 | PAIRED | ILLUMINA |
|  | 3 | Illumina Genome Analyzer II | C57BL/6 | 2~3 months | WT | Bone marrow | SP-KSL-CD150+CD48- | HSC_13 | SRX196449 | PAIRED | ILLUMINA |
|  |  |  |  |  |  |  |  | HSC_14 | SRX196448 | PAIRED | ILLUMINA |
|  |  |  |  |  |  |  |  | HSC_15 | SRX196447 | PAIRED | ILLUMINA |
| CMP | 1 | Illumina HiSeq 2000 | C57BL/6 | 6-12 weeks | WT | Bone Marrow | Lin− Sca-1− c-Kit+ CD150− CD34+ FcγRlo | CMP_1 | SRX1418300 | PAIRED | ILLUMINA |
|  |  |  |  |  |  |  |  | CMP_2 | SRX1418301 | PAIRED | ILLUMINA |
|  |  |  |  |  |  |  |  | CMP_3 | SRX1418302 | PAIRED | ILLUMINA |
|  |  |  |  |  |  |  |  | CMP_4 | SRX1418303 | PAIRED | ILLUMINA |
|  |  |  |  |  |  |  |  | CMP_5 | SRX1418304 | PAIRED | ILLUMINA |
| GMP | 1 | Illumina HiSeq 2000 | C57BL/6 | 6-12 weeks | WT | Bone Marrow | Lin− Sca-1− c-Kit+ CD150− CD34+ FcγR+ | GMP_1 | SRX1418309 | PAIRED | ILLUMINA |
|  |  |  |  |  |  |  |  | GMP_2 | SRX1418310 | PAIRED | ILLUMINA |
|  |  |  |  |  |  |  |  | GMP_3 | SRX1418311 | PAIRED | ILLUMINA |
|  |  |  |  |  |  |  |  | GMP_4 | SRX1418312 | PAIRED | ILLUMINA |
|  |  |  |  |  |  |  |  | GMP_5 | SRX1418313 | PAIRED | ILLUMINA |
|  | 2 | Illumina HiSeq 2000 | C57BL/6 |  | WT | Bone Marrow | IL-7Rα-, Lin- ,Sca-1-, c-Kit+, Cd34+, FcγRII/III-high | GMP_6 | SRX857070 | SINGLE | ILLUMINA |
|  |  |  |  |  |  |  |  | GMP_7 | SRX857074 | SINGLE | ILLUMINA |
|  |  |  |  |  |  |  |  | GMP_8 | SRX857078 | SINGLE | ILLUMINA |
|  |  |  |  |  |  |  |  | GMP_9 | SRX857082 | SINGLE | ILLUMINA |
|  | 3 | Ion Torrent Proton | C57BL/6 | Δ | WT | Bone Marrow | lineage- c-Kit+ Sca-1-, FcγR+ CD34+ | GMP_10 | SRX531209 | SINGLE | ION_TORRENT |
|  |  |  |  |  |  |  |  | GMP_11 | SRX531210 | SINGLE | ION_TORRENT |
|  |  |  |  |  |  |  |  | GMP_12 | SRX531213 | SINGLE | ION_TORRENT |
| Monocyte | 1 | Illumina HiSeq 2001 | C57BL/7 | 6 wk of age | WT | Bone Marrow | CD11b+ CD45high CD115+ Ly6G- | Monocyte_1 | SRX275697 | PAIRED | ILLUMINA |
|  |  |  |  |  |  |  |  | Monocyte_2 | SRX275698 | PAIRED | ILLUMINA |
|  |  |  |  |  |  |  |  | Monocyte_3 | SRX275699 | PAIRED | ILLUMINA |

**Suppl. Table 3. Data source of mouse HSC, CMP, GMP, and monocyte detected by our laboratory**

| **Cell_Type** | **Mouse Strain** | **Mouse Genotype** | **BioSample accession** |
| --- | --- | --- | --- |
| HSC | C57BL/6 | WT | SAMN27402797 |
| CMP | C57BL/6 | WT | SAMN27402912 |
| GMP | C57BL/6 | WT | SAMN27402913 |
| Monocyte | C57BL/6 | WT | SAMN27402952 |

**Suppl. Table 4. Data of mouse CMP and GMP ATAC-seq**

| **Cell_Type** | **Mouse Strain** | **PMID** | **Mouse Genotype** | **SRX** |
| --- | --- | --- | --- | --- |
| CMP | C57BL/6 | [25103404](https://www.ncbi.nlm.nih.gov/pubmed/25103404) | WT | SRX667510 |
| GMP | C57BL/6 | [25103404](https://www.ncbi.nlm.nih.gov/pubmed/25103404) | WT | SRX667503 |

**Suppl. Table 5. Data source of human HSC, CMP, GMP, and monocyte in different laboratories**

| **Cell_Type** | **SRX** |
| --- | --- |
| HSC | SRX1363185 |
|  | SRX1363191 |
|  | SRX1363199 |
|  | SRX1363209 |
| CMP | SRX1363183 |
|  | SRX1363189 |
|  | SRX1363197 |
| GMP | SRX1363184 |
|  | SRX1363190 |
|  | SRX1363198 |
| Monocyte | SRX1363187 |
|  | SRX1363193 |
|  | SRX1363202 |
| Acute monocyte leukemia | SRX693065 |
|  | SRX693070 |
|  | SRX693069 |
|  | SRX693068 |
|  | SRX693067 |
|  | SRX693066 |
|  | SRX693064 |
|  | SRX693062 |

**Suppl. Table 6. Regulated genes of glucose metabolism during HSC, CMP, GMP, and monocyte differentiation.**

|  | Pathway | ID | Input | Gene ID |
| --- | --- | --- | --- | --- |
| CMP vs HSC-upregulated | Oxidative phosphorylation | mmu00190 | 11 | 66152\|70383\|407785\|12867\|70316\|11944\|11951\|595136\|11947\|66108\|333182 |
|  | Glycolysis / Gluconeogenesis | mmu00010 | 7 | 212032\|14433\|56012\|18770\|68401\|68738\|18648 |
|  | Pentose and glucuronate interconversions | mmu00040 | 5 | 394435\|11677\|394433\|94284\|102448 |
| CMP vs HSC-downregulated | Glycolysis / Gluconeogenesis | mmu00010 | 20 | 11670\|60525\|110695\|11676\|74551\|319625\|72157\|14121\|353204\|11669\|103988\|16832\|16833\|67689\|11522\|56421\|72535\|13807\|12183\|15275 |
|  | Fructose and mannose metabolism | mmu00051 | 12 | 170768\|75540\|11676\|56421\|218138\|14121\|353204\|234730\|20322\|270198\|11997\|15275 |
|  | Pyruvate metabolism | mmu00620 | 12 | 60525\|17436\|68977\|74551\|18563\|11669\|52815\|16833\|16832\|72535\|110695\|100705 |
|  | Pentose phosphate pathway | mmu00030 | 7 | 11676\|56421\|14380\|353204\|14121\|19895\|72157 |
| GMP vs CMP-upregulated | Glycolysis / Gluconeogenesis | mmu00010 | 9 | 212032\|14121\|14751\|18648\|16833\|319625\|67689\|72535\|16832 |
|  | Starch and sucrose metabolism | mmu00500 | 8 | 212032\|232714\|74185\|22235\|14751\|394432\|14936\|110095 |
|  | Pentose phosphate pathway | mmu00030 | 5 | 14121\|75456\|66646\|110208\|14751 |
|  | Pyruvate metabolism | mmu00620 | 4 | 72535\|17436\|16833\|16832 |
| GMP vs Monocyte-upregulated | Amino sugar and nucleotide sugar metabolism | mmu00520 | 10 | 15212\|12763\|22235\|26384\|12764\|67883\|109754\|15277\|72017\|14583 |
|  | Pentose and glucuronate interconversions | mmu00040 | 8 | 11671\|11677\|22235\|71755\|11669\|394435\|394433\|394432 |
| GMP vs Monocyte-downregulated | Oxidative phosphorylation | mmu00190 | 45 | 76429\|67942\|66043\|11949\|66416\|11947\|74776\|12858\|225887\|69875\|66594\|27060\|333182\|12857\|67003\|66091\|67273\|66925\|66144\|67895\|75406\|22272\|67126\|68197\|66152\|67264\|12866\|67184\|70316\|66916\|11957\|11951\|11950\|226139\|69802\|17995\|226646\|407785\|84682\|11946\|228033\|22273\|68349\|28080\|68342 |
|  | Carbon metabolism | mmu01200 | 40 | 21351\|20917\|18642\|18641\|72141\|18648\|20425\|11409\|257635\|75456\|107272\|212032\|21991\|74147\|14433\|93747\|66925\|14751\|14194\|11429\|104776\|73724\|434437\|66904\|227095\|67834\|66171\|18563\|17448\|14121\|15275\|13807\|13806\|269951\|108037\|11674\|21881\|56451\|67078\|76282 |
|  | Pentose phosphate pathway | mmu00030 | 11 | 21351\|66171\|18641\|232449\|21881\|18642\|14751\|14121\|72157\|75456\|11674 |
|  | Pyruvate metabolism | mmu00620 | 11 | 68977\|18563\|17448\|76238\|14194\|52815\|16832\|16828\|100705\|110695\|66204 |
|  | Citrate cycle (TCA cycle) | mmu00020 | 9 | 20917\|67834\|66925\|18563\|17448\|14194\|11429\|56451\|269951 |
